# 3D DLP Fabrication of Electrically Conductive Polypyrrole Nanocomposites

**DOI:** 10.1002/smsc.70422

**Published:** 2026-10-07

**Authors:** Silas C. Eiden, Robert F. Sojka, Christian Delavier, Sebastian Grieger, Jana Zaumseil, Eva Blasco

**Affiliations:** ^1^ Institute for Molecular Systems Engineering and Advanced Materials (IMSEAM) Heidelberg University Heidelberg Germany; ^2^ Institute for Physical Chemistry Heidelberg University Heidelberg Germany

**Keywords:** additive manufacturing, conjugated polymers, nanoparticles, vat photopolymization

## Abstract

Conjugated polymers such as polypyrrole (PPy) combine high electronic conductivity with good biocompatibility, making them attractive for flexible electronics or biomedical applications. However, their limited processability remains a major challenge, motivating the exploration of advanced fabrication strategies such as additive manufacturing. Among these, light‐based 3D printing offers high resolution and geometric freedom. However, its compatibility with strong absorbing and poor soluble materials such as PPy remains limited. Here, we report a new strategy integrating presynthesized, defined PPy nanoparticles into photocurable inks for digital light processing (DLP) 3D printing. Four distinct PPy nanoparticles have been synthesized via chemical oxidation polymerization using FeCl_3_ or APS as oxidants and SDS or DBSA as surfactants in aqueous media. The resulting materials have been characterized in depth in terms of their structural, optical, morphological, and electrical properties. Stable inks containing 3.0–5.0 wt% PPy nanoparticles enable DLP printing at 405 nm, yielding single‐ and multilayer 3D structures with high fidelity, rapid curing and electrical conductivities up to 3 × 10^−2^ S cm^−1^. This work establishes a new route for direct incorporation of PPy into inks for DLP printing, opening new opportunities for the additive manufacturing of electrically conductive polymer materials.

## Graphical abstract

A new approach enables the incorporation of polypyrrole nanoparticles into photocurable inks for DLP 3D printing. The resulting materials combine printability, structural fidelity, and electrical conductivity, demonstrating the potential of light‐based additive manufacturing for producing conductive polymer structures.

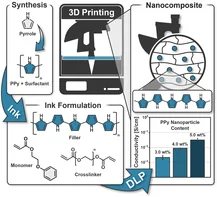

## 1 Introduction

Organic electronics, flexible sensors, bio interfaces, and energy‐conversion devices increasingly rely on lightweight, mechanically compliant, and electrically active materials [1, 2]. Conjugated polymers (CPs) such as polyaniline (PANI), polypyrrole (PPy), poly(3‐hexylthiophene) (P3HT), poly(3,4‐ethylenedioxythiophene) (PEDOT), and other donor–acceptor copolymers have emerged as key candidates for such applications due to their unique combination of electronic functionality and structural versatility [3, 4, 5, 6, 7]. Their π‐conjugated backbones create extended domains of delocalized π‐electrons that enable intrinsic charge transport [8, 9]. Although their charge‐carrier mobility and conductivity remain inferior to those of inorganic or metallic counterparts, CPs offer distinct advantages, including synthetically tunable electronic properties, versatile mechanical behavior, straightforward fabrication, and good environmental stability [3, 10, 11].

PPy has attracted significant attention owing to its high electrical conductivity of 1–100 S cm^−1^, biocompatibility, and synthetic accessibility [12, 13, 14]. PPy can be synthesized through several complementary routes, including electrochemical polymerization, photochemical polymerization, and chemical oxidative polymerization, each of which distinctly influences the resulting electronic properties [15, 16, 17, 18]. Electrochemical methods enable precise control over film thickness, doping level, and deposition site, making them particularly suitable for coatings and device integration [19]. Photochemical routes offer spatial control through light‐mediated initiation, while chemical oxidative polymerization provides a rapid, scalable, and straightforward pathway to multigram quantities of bulk material [20, 21, 22]. In chemical oxidative polymerization, the electrical, mechanical, and physicochemical properties of PPy, as well as its molecular structure and morphology, can be tuned by adjusting reaction parameters such as oxidant type, oxidant‐to‐monomer ratio, temperature, and solvent environment [15]. In addition, the incorporation of anionic surfactants with linear alkyl chains, such as sodium dodecyl sulfate (SDS) and 4‐dodecylbenzene sulfonic acid (DBSA), can serve simultaneously as soft templates and codopants [15, 23, 24, 25, 26, 27]. These additives reduce interchain π–π interactions and improve dispersibility, although often at the expense of electrical conductivity due to increased charge‐transport distances [15]. Despite its synthetic versatility, the intrinsic rigidity of the conjugated backbone leads to poor solubility, limited mechanical robustness, and restricted melt processability. These constraints complicate the fabrication of complex three‐dimensional (3D) architectures and motivate the development of advanced processing strategies [28, 29, 30, 31, 32, 33].

Interest has increasingly shifted toward additive manufacturing (AM), with notable progress in producing PPy‐containing materials using techniques such as fused deposition modeling (FDM), inkjet printing, and direct ink writing (DIW) [34, 35, 36, 37, 38, 39]. Within the AM landscape, light‐based printing techniques such as stereolithography (SLA) and digital light processing (DLP) have gained particular attention due to their high spatial resolution, rapid curing, and ability to fabricate intricate architecurally complex 3D geometries [40, 41, 42, 43, 44, 45, 46]. However, light‐based processes rely on controlled light penetration and efficient photopolymerization, both of which are highly sensitive to the optical properties of the inks [47]. Because PPy strongly absorbs in the ultraviolet and visible regions of the electromagnetic spectrum (UV/vis), its presence reduces curing depth, slows the polymerization rate, and ultimately compromises the printing performance [34]. To circumvent these challenges, two main approaches have been reported: The first involves postprinting polymerization of PPy in a stepwise process by immersing the printed structures in a monomer solution followed by polymerization in an oxidant solution, thereby generating PPy within the polymer matrix [48, 49]. However, this strategy presents several limitations, including additional processing steps after printing and limited control over the distribution and properties of the resulting conductive phase. The second approach relies on the in situ photopolymerization of PPy during the printing process by directly incorporating pyrrole and the oxidant species into the ink formulation [50, 51]. Despite enabling simultaneous printing and polymer formation, the materials obtained through this method typically exhibit low electrical conductivities (around 10^−5^ S cm^−1^), limited printing resolution, and material stability [51]. Consequently, alternative strategies are required to overcome the intrinsic incompatibility between PPy formation and light‐based photopolymerization processes. In particular, the use of preformed PPy dispersions represents a promising yet underexplored route in light‐based 3D printing for integrating conductive domains while preserving printability.

Herein, we present an approach that integrates defined PPy nanoparticles as conductive fillers into acrylate‐based inks for the one‐step fabrication of electrically conductive materials with high resolution using DLP printing (Figure 1). To systematically investigate the influence of the filler, four distinct PPy‐based nanoparticles were synthesized and comprehensively characterized. We found that the incorporation of surfactants and the choice of the oxidant play an important role in the resulting PPy morphology, conductivity, and dispersability and, therefore, printability. The corresponding PPy nanoparticle‐containing inks were optimized for light‐based AM, and the resulting conductive DLP‐printed materials were thoroughly evaluated. Importantly, robust characterization protocols were established for printed structures across different length scales, ranging from 2D single layers to fully 3D architectures. We demonstrate that complex structures with finely resolved feature sizes can be directly printed without postprocessing while achieving electrical conductivities of up to 3 × 10^−2^ S cm^−1^. These values rank among the highest conductivities reported in the literature for PPy‐based materials fabricated via light‐based 3D printing techniques.

**FIGURE 1:**
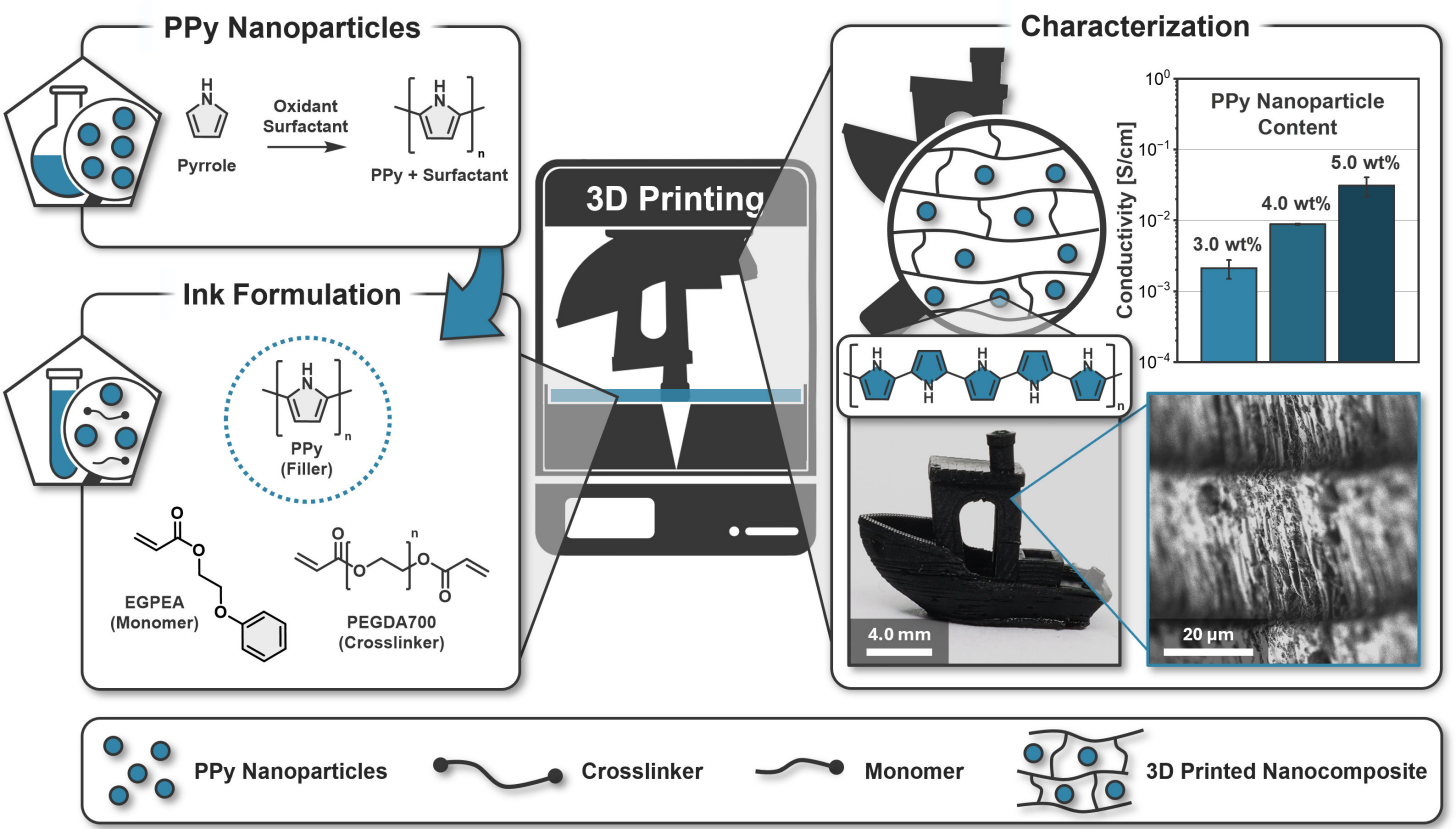
Workflow for the 3D DLP printing of PPy nanocomposites. Synthesis of PPy nanoparticles (fillers) by chemical oxidative polymerization with a combination of oxidant and surfactant: **PPy‐Fe‐SDS**, **PPy‐Fe‐DBSA**, **PPy‐APS‐SDS**, and **PPy‐APS‐DBSA**. Ink formulation containing monomer **EGPEA**, crosslinker **PEGDA700**, **PPy filler**, and **BAPO** as a photoinitiator. DLP printing of nanodispersions containing **PPy filler** at a 405 nm wavelength. Analysis of 3D‐printed materials with embedded PPy nanoparticles reveals high conductivity and high resolution. Nanoparticle incorporation into the printed material could be revealed and visualized by scanning electron microscopy (SEM) imaging.

## 2 Results and Discussion

### 2.1 Printable Formulations Incorporating Defined PPy Nanoparticles

#### 2.1.1 Synthesis of PPy Nanoparticles

To facilitate the incorporation of PPy in a formulation for DLP 3D printing, we first prepared a series of PPy nanoparticles via chemical oxidative polymerization (Scheme 1) [52]. To this aim, we selected two common oxidizing agents (**X**), i.e., iron (III), chloride (Fe), and ammonium persulfate (APS), and tested different surfactants (**Y**), SDS and DBSA (for synthetic details, see Section S1). The four resulting materials (**PPy‐X‐Y**), **PPy‐Fe‐SDS**, **PPy‐Fe‐DBSA**, **PPy‐APS‐SDS**, and **PPy‐APS‐DBSA,** were obtained as black powders.

**SCHEME 1:**
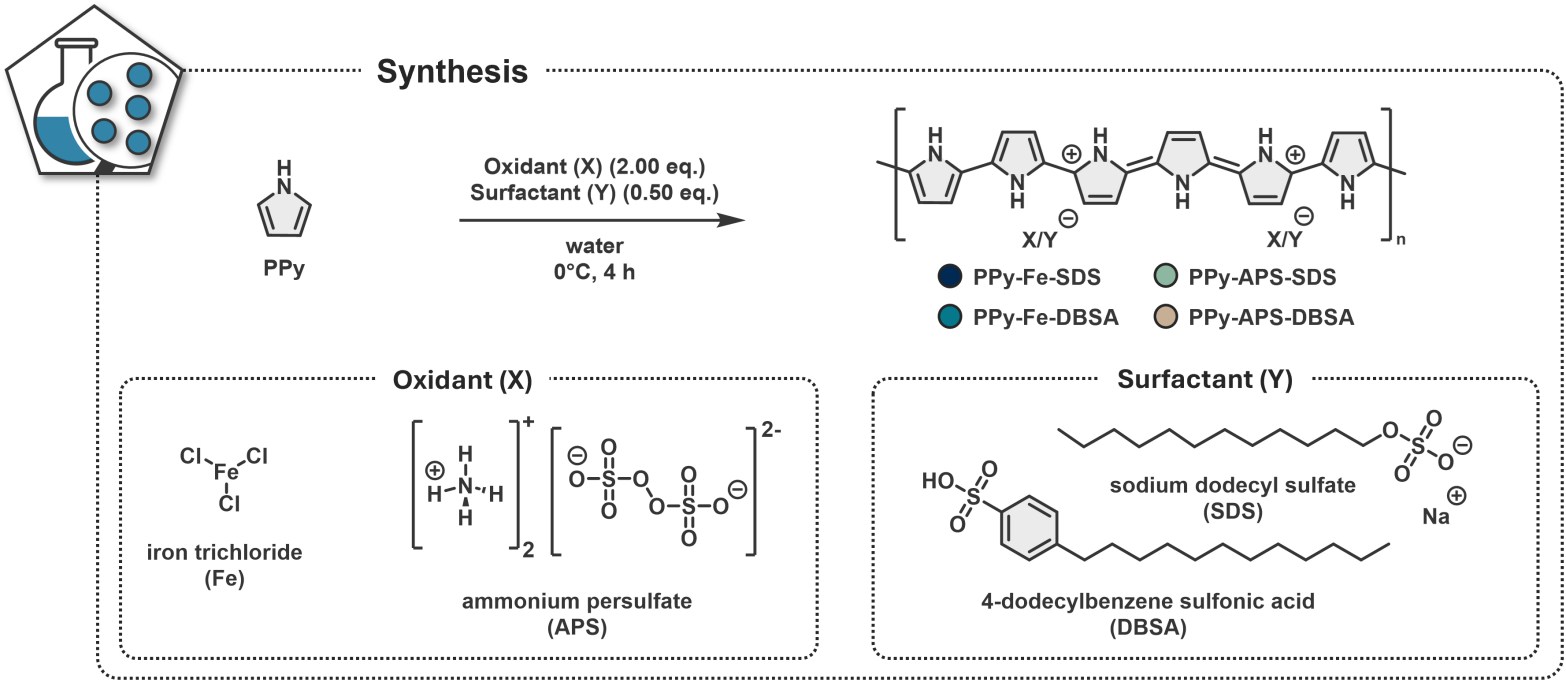
Chemical oxidative polymerization in water. By the combination of the two different oxidants FeCl_3_ and APS with the surfactants SDS and DBSA, four PPy nanoparticles, **PPy‐Fe‐SDS**, **PPy‐Fe‐DBSA**, **PPy‐APS‐SDS**, and **PPy‐APS‐DBSA**, were obtained.

As a next step, the structural and electronic features of the synthesized PPy nanoparticles were investigated. Raman spectroscopy was first employed to verify the formation of PPy (Figure 2A) (Section S2). In particular, four main vibrational regions can be distinguished: deformation vibrations $\left({\tilde{\delta }}_{\left(C-C\right)}\right)$ of polarons (963–985 cm^−1^) and bipolarons (926–927 cm^−1^), deformation vibrations $\left({\tilde{\delta }}_{\left(C-H\right)}\right)$ of polarons (1050–1056 cm^−1^) and bipolarons (1079–1084 cm^−1^), ring stretching vibrations (*ṽ*_(C─C)_) (1336–1377 cm^−1^) and stretching vibration (*ṽ*_(C═C)_) of the polarons (1575–1600 cm^−1^) and bipolarons (1600–1632 cm^−1^), which are in agreement with previous literature [26, 41, 48, 49, 53]. From the comparison of polaron and bipolaron signal intensities, a qualitative comparison of doping levels is possible [26]. Herein, APS‐oxidized **PPy‐APS‐SDS** and **PPy‐APS‐DBSA** exhibited higher oxidation levels, suggested by the higher polaron/bipolaron ratio of the C═C stretching vibrations [16, 52, 54]. FT‐IR spectroscopy (Figure 2B) further supports these findings, showing characteristic pyrrole ring vibrations around 1540, 1455, and 1303 cm^−1^, respectively [41, 49, 55]. Carbonyl stretching bands around 1710 cm^−1^ for **PPy‐APS‐SDS** and **PPy‐APS‐DBSA** indicate the formation of carbonyl defects induced by the stronger oxidant APS (Section S3) [56, 57].

**FIGURE 2:**
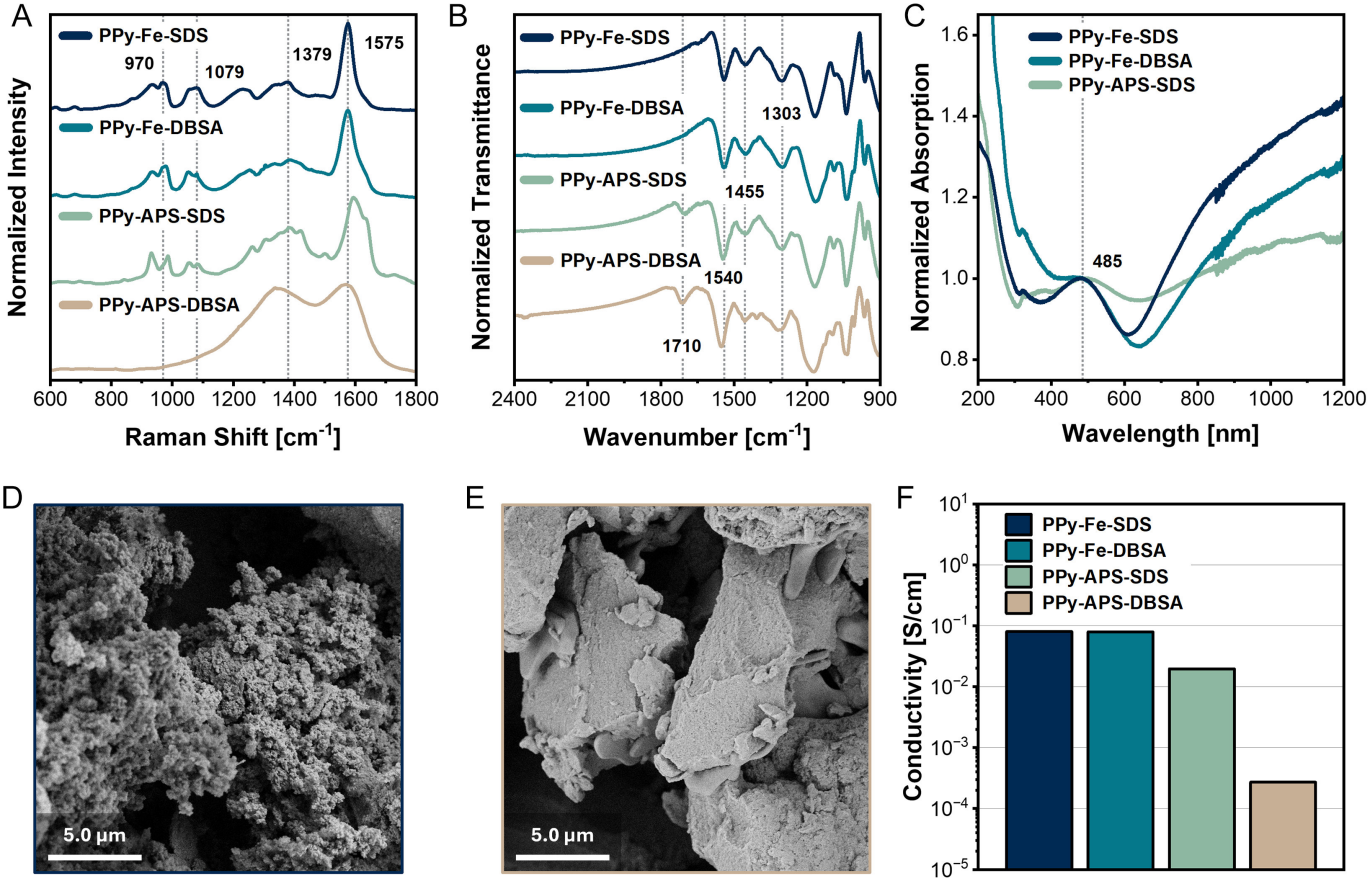
Characterization of PPy nanoparticles **PPy‐Fe‐SDS**, **PPy‐Fe‐DBSA**, **PPy‐APS‐SDS**, and **PPy‐APS‐DBSA**: (A) Normalized Raman spectra of PPy filler pellets displayed with a manual offset for comparison. (B) Normalized FT‐IR spectra of PPy/KBr pellets displayed with a manual offset for comparison. (C) Absorption spectra of aqueous dispersions of PPy nanoparticles normalized to the 485 nm polaron band. PPy‐APS‐DBSA is not displayed due to sedimentation and, hence, insufficient absorption. (D) SEM image of **PPy‐Fe‐SDS**. (E) SEM image of **PPy‐APS‐DBSA**. (F) Electrical conductivity of PPy nanoparticles.

The optical properties of the PPy nanoparticles were also characterized. Upon ultrasonication in water, dark but translucent colloidal suspensions were obtained for **PPy‐Fe‐SDS** and **PPy‐APS‐SDS** (Section S4). In contrast, dispersions of **PPy‐Fe‐DBSA** and **PPy‐APS‐DBSA** formed an inhomogeneous mixture with rapid particle sedimentation. These observations indicate that the use of SDS as a surfactant significantly improves nanoparticle dispersibility in water. Accordingly, **PPy‐Fe‐SDS** and **PPy‐APS‐SDS** exhibited more intense and broader absorption bands compared to their DBSA‐based counterparts, extending across the measured spectral range and being consistent with literature reports (Figure 2C). Distinct absorption peaks at 485 nm can be attributed to polaron transitions, while bipolaron‐related absorption extends into the near‐infrared (NIR) above 1200 nm [26]. Comparison of these polaron and bipolaron bands suggests higher oxidation levels for **PPy‐Fe‐SDS** compared to **PPy‐Fe‐DBSA**. However, a comparison of the APS‐oxidized PPy nanoparticles was not possible due to the lack of stability of the dispersions.

Morphological and particle size analyses of the PPy nanoparticles were also conducted to evaluate their dispersibility, a key factor for printable inks. In this regard, SEM imaging revealed submicrometer granular clusters composed of smaller nanoparticles for **PPy‐Fe‐SDS**, **PPy‐Fe‐DBSA,** and **Fe‐APS‐SDS,** while **PPy‐APS‐DBSA** formed large agglomerates (Figure 2D,E, respectively). Statistical analysis of SEM images of diluted nanoparticle dispersions allowed the evaluation of the average particle size resulting in 58.1 nm (**PPy‐Fe‐SDS**), 42.2 nm (**PPy‐Fe‐DBSA**), and 55.0 nm (**PPy‐APS‐SDS**), respectively (Section S5). Dynamic light scattering (DLS) further confirmed the formation of submicrometer clusters for the three PPy nanoparticles. Together, these results highlight that both surfactant choice and oxidant type strongly influence particle morphology and, hence, dispersibility.

The electrical conductivity of the obtained materials was measured using the two‐point probe method (Section S6). **PPy‐Fe‐SDS** and **PPy‐Fe‐DBSA** exhibited the highest conductivities, reaching approximately 8.0 × 10^−2^ S cm^−1^, followed by **PPy‐APS‐SDS** (2.0 × 10^−2^ S cm^−1^). Conductivities two orders of magnitude smaller were found for **PPy‐APS‐DBSA** (2.7 × 10^−4^ S cm^−1^) (Figure 2F). These findings align well with literature reports, which show that higher conductivities are typically achieved with PPy of smaller particle sizes and moderate oxidation levels synthesized using FeCl_3_ as an oxidant [15, 26].

#### 2.1.2 Incorporating PPy Nanoparticles as Fillers in Printable Formulations

Next, the PPy nanoparticles (**PPy‐Fe‐SDS**, **PPy‐Fe‐DBSA**, **PPy‐APS‐SDS,** and **PPy‐APS‐DBSA)** were incorporated into a photocurable acrylate formulation, enabling their use as functional fillers in light‐based 3D printing (Figure 3). Ethylene glycol phenyl ether acrylate (**EGPEA**) (80.6 wt%) was chosen as the monomer, promoting π–π interactions between the phenyl groups and the polymer, an effect previously reported for PANI particles [58]. Poly(ethylene glycol) diacrylate (**PEGDA700**) (15.4 wt%) was used as a bifunctional acrylate crosslinker, providing flexibility to the printed material and reducing hydrophobicity of the ink formulation. Phenyl (2,4,6‐trimethylbenzoyl) phosphine oxide (**BAPO**) (1.0 wt%) was used as a photoinitiator for the DLP printing at 405 nm.

**FIGURE 3:**
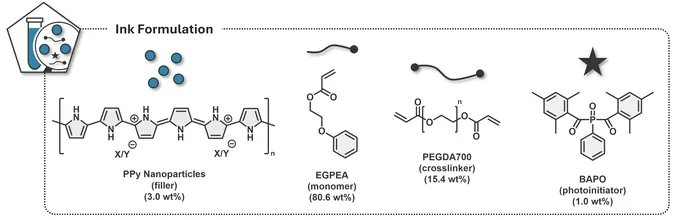
Ink formulation for the implementation of 3.0 wt% of **PPy‐Fe‐SDS**, **PPy‐Fe‐DBSA**, **PPy‐APS‐SDS**, and **PPy‐APS‐DBSA** into photocurable inks for DLP printing, respectively. **EGPEA** was used as a monomer, **PEGDA700** as a crosslinker and **BAPO** as a photoinitiator for the printing at a 405 nm wavelength.

The printability of the four formulations was evaluated using a commercial DLP printer using an LED source emitting at 405 nm, corresponding to a local minimum of PPy absorption (see Figure 2C). The corresponding Jacobs working curves were generated for all the formulations, providing penetration depth (*D*_p_) of the curing light and the minimum energy required for polymerization (critical curing energies: *E*_c_) (Section S7). The incorporation of a 3.0 wt% filler strongly reduced the light penetration depth from 275 µm for the pristine ink to 68–83 µm, with the lowest values observed for SDS‐bearing **PPy‐Fe‐SDS** and **PPy‐APS‐SDS**. Critical curing energies were also affected, with DBSA‐containing inks remaining comparable to the pristine formulation (**PPy‐Fe‐DBSA**: 7.06 mJ cm^−2^ and **PPy‐APS‐DBSA**: 5.75 mJ cm^−2^), while SDS‐containing inks required markedly higher energies (**PPy‐Fe‐SDS**: 26.2  mJ cm^−2^ and **PPy‐APS‐SDS**: 47.0  mJ cm^−2^), which is consistent with their absorption at 405 nm. Despite these differences, all inks containing 3.0 wt% PPy nanoparticles exhibited reliable curing at a light intensity of 18 mW cm^−2^, with exposure times ranging from 1 to 20 s. Increasing the filler content of **PPy‐Fe‐DBSA** and **PPy‐APS‐SDS** above 3.0 wt% led to inks with a strong tendency toward agglomeration, unsuitable for DLP printing.

As **PPy‐Fe‐SDS** was identified as the most promising filler due to its good dispersibility and high conductivity, its printability was investigated in more detail. A stepwise increase in filler content showed that inks containing up to 6.0 wt% could still cure effectively, although as expected, light penetration decreased and *E*_c_ rose from 53.3 mJ cm^−2^ (4.0 wt%) to 77.5 mJ cm^−2^ (6.0 wt%) (Table S2). Filler loadings exceeding 6.0 wt% caused particle sedimentation and poor material stability, defining the upper limit for the formulation.

### 2.2 From 2D to 3D DLP Printing of PPy‐Containing Architectures

As DLP is a layer‐by‐layer technique, understanding how individual layers contribute to the final conductivity is essential for rational material design. Therefore, we began with a well‐defined 2D single layer and subsequently extended the approach to multilayer structures relevant for practical applications. First, 2D single‐layer squares (15 × 15 mm^2^) were printed with a thickness of 50 μm on silanized glass slides using the previously optimized inks (Figure 4A). The best‐defined layers were obtained using inks containing 3.0 wt% **PPy‐Fe‐DBSA** and **PPy‐APS‐SDS** as well as 3.0–5.0 wt% **PPy‐Fe‐SDS**, while inks containing 3.0 wt% **PPy‐APS‐DBSA** were unsuitable for the fabrication of defined single layers.

**FIGURE 4:**
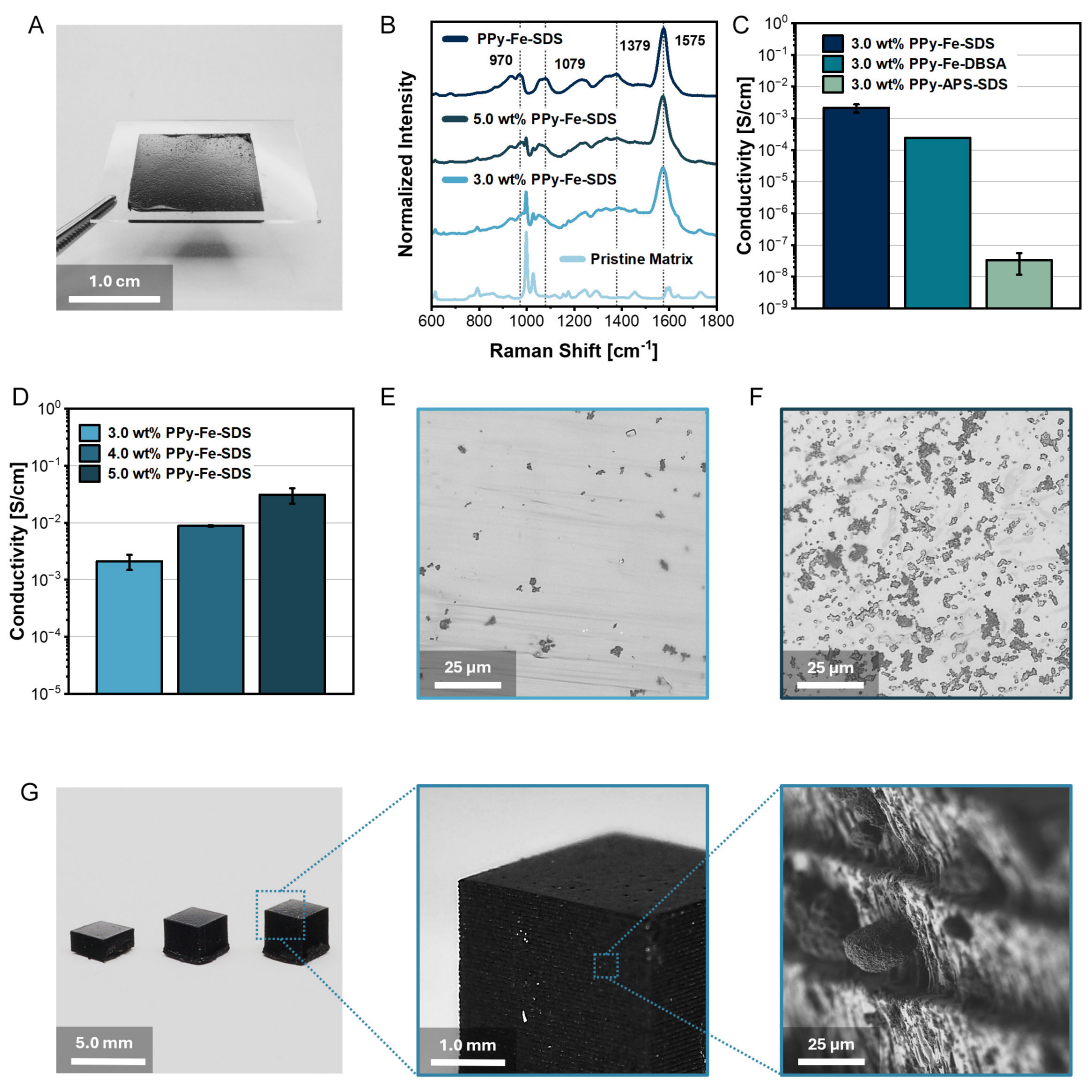
Characterization of DLP‐printed PPy‐containing composites: (A) Single layer DLP‐printed on silanized glass. (B) Normalized Raman spectra of respective single layers containing 3.0–5.0 wt% **PPy‐Fe‐SDS** compared to the pristine matrix and the pristine **PPy‐Fe‐SDS** nanomaterial. Spectra plotted with a manual offset for comparison. (C) Electrical conductivity of DLP‐printed single layers of inks containing 3.0 wt% **PPy‐Fe‐SDS**, **PPy‐Fe‐DBSA**, and **PPy‐APS‐SDS** measured with the four‐point probe method, respectively. (D) Electrical conductivity of DLP‐printed single layers containing 3.0–5.0 wt% of **PPy‐Fe‐SDS** measured with the four‐point probe method. (E) Optical microscope image of a single layer containing 3.0 wt% **PPy‐Fe‐SDS**. (F) Optical microscope image of a single layer containing 5.0 wt% **PPy‐Fe‐SDS**. (G) Cubes of increasing number of layers (left to right: 1.5, 2.0, and 2.5 mm) printed with inks containing 3.0 wt% **PPy‐Fe‐SDS** (left). Macroscopic image of printed cubes (middle). SEM image of printed layers (right) showing **PPy‐Fe‐SDS** nanoparticles and larger agglomerates on the surface.

The incorporation of PPy nanoparticles was unambiguously confirmed by Raman and FT‐IR spectroscopy. In the Raman spectra, characteristic vibrational modes of PPy increased in intensity around 970, 1079, 1379, and 1575 cm^−1^, when increasing the PPy content in the ink, while prominent signals of the monomer **EGPEA** around 1000 cm^−1^ decreased. (Figure 4B and Section S8). Similarly, the FT‐IR spectra revealed enhanced stretching vibration (*ṽ*_(C═C)_) at 1540 cm^−1^ and pyrrole‐associated bands at 925, 885, and 851 cm^−1^. Double‐bond conversion measurements confirmed that the incorporation of **PPy‐Fe‐SDS** up to 5.0 wt% does not significantly impair the polymerization of the acrylate matrix, with conversion remaining comparable (around 90%) to that of the unfilled formulation (Section S9).

The electrical conductivity of the single layers was measured by a four‐point probe method (Section S10). Among all the formulations, the printed layers using inks containing 3.0 wt% **PPy‐Fe‐SDS** showed the highest conductivity of 2.1 × 10^−3^ S cm^−1^ followed by **PPy‐Fe‐DBSA** with 2.4 × 10^−4^ S cm^−1^ (Figure 4C). In contrast, printed layers containing 3.0 wt% **PPy‐APS‐SDS** showed very low electrical conductivity at 3.3 × 10^−8^ S cm^−1^, indicating that the filler particles do not reach the percolating threshold due to their size or inhomogeneous distribution. Importantly, measurements of single layers using the same setup applying the two‐point probe method displayed comparable electrical conductivities and thus a negligibly small contact resistance compared to the resistance of the material.

Increasing the **PPy‐Fe‐SDS** content above 3.0 wt% resulted in a marked conductivity enhancement, rising from 8.8 × 10^−3^ S cm^−1^ (4.0 wt%) to 3.1 × 10^−2^ S cm^−1^ (5.0 wt%) (Figure 4D). This behavior indicates percolation‐driven behavior. Electrical impedance spectroscopy (EIS) revealed no significant contribution from ionic conduction observed under the investigated conditions (Section S11). Notably, the conductivities obtained for **PPy‐Fe‐SDS** nanocomposites rank among the highest conductivities reported for PPy‐containing composite materials fabricated using light‐based AM (Section S12) [41, 42, 48, 50, 51].

Optical microscopy images of printed single layers further supported this interpretation (Figure 4E,F as well as Section S13). With increasing filler loading, both the number and size of agglomerated PPy particles on the film surface became more pronounced. In addition to finely dispersed nanoparticles, larger conductive domains were observed, which function as conductive islands within the polymer matrix.

After establishing the methodology for 2D layers, we extended the approach to multilayer structures to investigate their influence on the final properties and to exploit the unique layer‐by‐layer capabilities of DLP 3D printing. First, 3D cubes of increasing numbers of layers were printed using inks containing **PPy‐Fe‐SDS**. Herein, well‐defined 3D structures could be obtained with inks containing up to 3.0 wt% **PPy‐Fe‐SDS** (Figure 4G). SEM imaging of the printed structure revealed the incorporation of **PPy‐Fe‐SDS** particles of different sizes inside and on the surface of the printed structure (Section S14). Moreover, a higher concentration of **PPy‐Fe‐SDS** particles is found on the sides facing the layers, displaying a higher surface area compared to the less covered and smooth top of the printed structure. Due to the absorption of **PPy‐Fe‐SDS** in the ink, the layers progressively shrink from the illuminated side toward the bottom, producing a saw‐blade profile in the cross‐section of the layers. Consequently, further aggregation of PPy particles due to higher filler loadings reduces curing further and leads to poor first‐layer and interlayer adhesion. Thermogravimetric analysis revealed no change in stability upon the incorporation of **PPy‐Fe‐SDS** into the matrix (Section S15).

The electrical conductivity in multilayer printed structures was systematically investigated by fixating the structure between two gold‐coated glass slides (Section S16). This configuration enabled conductivity measurements parallel (*σ*_‖_) and perpendicular (*σ*_⟘_) to the printed layers. The in‐plane conductivity (*σ*_‖_) of multilayer structures was measured at 1.6 × 10^−4^ S cm^−1^, approximately one order of magnitude lower than that of the optimized single‐layer samples (2.1 × 10^−3^ S cm^−1^). Measurements performed perpendicular to the printed layers revealed a further decrease in conductivity (*σ*_⟘_ = 1.3 × 10^−6^ S cm^−1^), demonstrating anisotropic charge transport within the printed architecture. This anisotropy likely results from the layer‐by‐layer processing method giving a gradient in **PPy‐Fe‐SDS** particle concentration inside each printed layer due to sedimentation of conductive filler particles during the printing process, ultimately enhancing the contact resistance between the layers [59].

Finally, the printability of the **PPy‐Fe‐SDS** ink was demonstrated through the fabrication of centimeter‐scale 3D structures with increasing geometric complexity and fine structural details (Section S17). Sunflower‐shaped objects printed with 3.0 wt% **PPy‐Fe‐SDS** exhibited high resolution and good feature fidelity despite some overpolymerization observed in initial burn‐in layers required to ensure sufficient adhesion to the print head during printing (Figure 5A). To further showcase the robustness of the formulation, complex geometries with pronounced overhangs, including chess pieces and Benchy models, were successfully fabricated without the use of support structures (Figure 5B,C). These results highlight the architectural freedom enabled by the conductive ink system.

**FIGURE 5:**
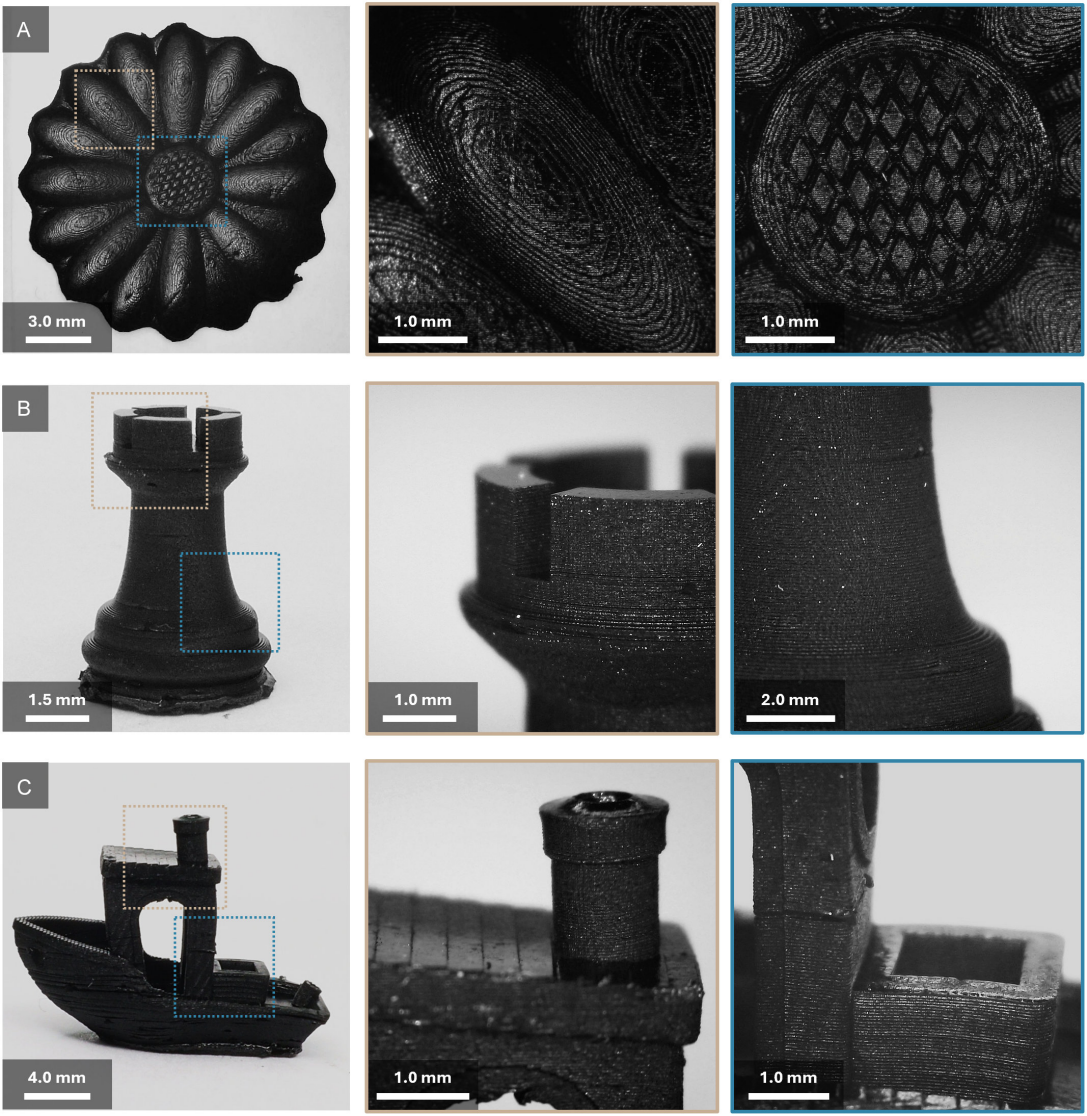
Overview of structures of increasing complexity DLP‐printed with a layer height of 50 μm from inks containing 3.0 wt% **PPy‐Fe‐SDS**: (A) Sunflower structure (CAD: 15.0 × 15.0 × 1.30 mm, measured: 15.2 × 15.3 × 1.38 mm). (B) Chess piece (CAD: 7.55 × 7.55 × 11.25 mm, measured: 7.57 × 7.61 × 11.3 mm). (C) Benchy structure (CAD: 15.1 × 7.81 × 12.0 mm, measured: 15.2 × 7.88 × 12.1 mm) with overhanging features printed without support.

## 3 Conclusion

In this work, we have synthesized four different PPy nanoparticles by chemical oxidative polymerization using a combination of an oxidant and additional surfactant to alter the morphology and dispersibility of the respective nanomaterials. The four PPy materials (**PPy‐Fe‐SDS**, **PPy‐Fe‐DBSA**, **PPy‐APS‐SDS**, and **PPy‐APS‐DBSA**) were characterized in depth. Raman spectroscopy revealed oxidation levels using FeCl_3_ as an oxidant, leading to favorable electrical conductivities of up to 8.0 × 10^−2^ cm^−1^ for neat **PPy‐Fe‐SDS**. In contrast, APS‐oxidized samples showed lower conductivity levels. Furthermore, SEM analysis revealed distinct morphological differences: When oxidized with FeCl_3_, PPy yielded fine granular particles in the presence of either SDS or DBSA. When oxidized with APS, PPy was finely granular with SDS but formed larger, sheet‐like morphologies with DBSA. These morphological variations significantly influence dispersibility and subsequent ink formulation performance.

The incorporation of **PPy‐Fe‐DBSA** and **PPy‐APS‐SDS** into a photocurable formulation enabled the light‐based printing of defined single layers with a filler loading of 3.0 wt%. Among the investigated systems, **PPy‐Fe‐SDS** exhibited superior ink compatibility, allowing stable formulations with up to 5.0 wt% filler loading. The electrical conductivity of the printed materials was characterized by using both two‐ and four‐point probe measurements on single‐layer and multilayer cube structures. The highest conductivity of 3.1 × 10^−2^ S cm^−1^ was achieved for single‐layer structures containing 5.0 wt% **PPy‐Fe‐SDS**. Furthermore, complex multilayer geometries with fine structural details, including chess pieces and Benchy models, were successfully fabricated.

In conclusion, this study demonstrates a successful strategy for 3D printing high‐resolution and electrically conductive structures through the incorporation of PPy nanoparticles as functional fillers. Our results show that reliable photocuring and high electrical conductivity in printed materials require the use of defined, oxidized PPy particles with a nanoscale morphology and surface functionalization with anionic surfactants. Future work will focus on extending this strategy to other functionally CPs and multimaterial systems, enabling broader applications in flexible electronics and bioelectronic devices.

## 4 Experimental Section

### 4.1 Materials

Pyrrole (98% Sigma‐Aldrich) was distilled under a nitrogen atmosphere and ambient pressure and stored at −19 °C until use. Iron(III) chloride (Fe) (97%), DBSA (≥95%), ammonium peroxydisulfate (APS) (≥98%), BAPO (97%), poly(ethylene glycol) diacrylate (PEGDA700) (*M*_n_ = 700 stabilized with 100 ppm 4‐methoxyphenol (MEHQ) and 300 ppm butylated hydroxytoluene (BHT)) and 3‐(trimethoxy‐silyl)propyl acrylate (92%) were acquired from Sigma‐Aldrich. Ethylene glycol phenyl ether acrylate (EGPEA) (>93%) was purchased from TCI Chemicals. SDS (98%) was obtained from BLDpharm. Solvents were purchased from Fisher Scientific and VWR Chemicals. All chemicals were used as received without further purification, unless indicated. Reagents sensitive to light were stored in the dark at 4 °C or −19 °C and handled under yellow‐light conditions.

### 4.2 Fabrication Procedures

**Dispersions of PPy** in inks for printing and in water for analysis were done by ultrasonication using an Elmasonic Easy 60 H in water for 60 min. Vials of the dispersion were directly placed at the center of the cavitation hotspot.

**PPy pellets** of vacuum‐dried, finely ground PPy (300 mg, neat) and PPy/KBr mixtures (0.1 mg PPy in 300 mg KBr) were prepared by pressing discs of 13.0 mm diameter using a hydraulic laboratory press applying a 10 t load for 5 min.

**PPy nanoparticle films** for SEM size analysis were prepared by drop‐casting PPy dispersions (0.5 mg/mL in water with SDS or DBSA, 5.0 mg/mL) onto single‐side‐polished, p‐doped (B) silicon substrates (525 ± 20 µm, Siegert Wafer) at 80 °C. Prior to fabrication, the substrates were cut to size and cleaned by sequential ultrasonication in isopropanol and acetone for 10 min each. After drop‐casting, the resulting films were immersed overnight in water (for SDS‐containing films) or a 1:1 water/ethanol mixture (for DBSA‐containing films), then dried under vacuum prior to SEM analysis.

**Silanized glass slides** were prepared by sonicating glass slides (Schott, 20 × 25 × 0.5 mm) in isopropanol and acetone for 10 min and dried under a stream of compressed nitrogen. The surface was activated for 30 min using a plasma cleaner (Harrick Plasma, Model PDC‐002). Subsequently, the glass slides were immersed in a solution of 3‐(trimethoxy‐silyl)propyl acrylate (4.0 × 10^−3^ M in toluene) for 18 h. After rinsing with toluene twice and once with acetone, the silanized glass slides were stored until use. Preparation as well as storage occurred under yellow‐light conditions.

**Fabrication of gold electrodes** was done by cleaning glass slides (Schott, 20 × 25 × 0.5 mm) by sonication using isopropanol and acetone for 10 min, respectively. Afterward, the cleaned glass slides were dried under a stream of compressed nitrogen. Afterward, the desired pattern of 100 nm gold above a 2.0 nm thick adhesive layer of chromium was thermally evaporated in a vacuum at a rate of 0.6 Å/s through a custom‐made shadow mask using a thermal evaporator (MB ProVap 3G, M. Braun Inertgas‐Systeme). Electrodes for electrical impedance measurements were fabricated by evaporation of 37.5 nm gold above 2.5 nm chromium on glass slides (Schott, 20 × 25 × 0.5 mm) and a circular glass substrate (*Ø* = 6 mm, thickness = 0.15 mm, Epredia), respectively.

### 4.3 Characterization Methods

**UV/vis spectroscopy** was conducted using a Jasco UV–vis–NIR V‐770 spectrophotometer in the spectral range of 200–1200 nm with 0.2 nm resolution and an integration time of 0.06 s. Data was exported in.csv format and processed using Origin 2024 10.1.0.178. Dispersions of PPy (0.05 mg/mL) in water were ultrasonicated for 1 h prior to the measurements.

**FT‐IR spectroscopy** of doped PPy samples embedded in KBr pellets was performed using a Thermo Scientific Nicolet iS5 FT‐IR spectrometer equipped with an iD1 Transmission Accessory, operating in transmission mode. The background was measured against a neat KBr pellet.

FT‐IR spectra of printed single layers were acquired using a Bruker LUMOS‐II FT‐IR microscope in the attenuated total reflectance (ATR) mode. FT‐IR spectra of ink formulations were measured using a Jasco FT/IR‐4600 spectrometer (ATR mode). All ATR measurements were referenced against air as the background. All spectra were recorded in the range of 600–4000 cm^−1,^ exported in.csv format, and processed using Origin 2023 10.0.0.154.

**Raman spectroscopy** of pressed pellets of PPy and printed single layers was performed on a Renishaw inVia confocal Raman microscope in backscattering configuration using a 50× long working distance objective (N.A. 0.5, Olympus) and a Nd:YAG (532 nm) laser in the range of 600–1800 cm^−1^. Calibration was performed with a silicon wafer to the signal at 520.6 cm^−1^ prior to each measurement. To minimize the influence of spot‐to‐spot variations, maps with >25 spectra were recorded and averaged using an integration time of 10 s per spectrum. Prebleaching (5 s) was applied prior to acquisition to reduce background photoluminescence.

**Scanning electron microscopy (SEM)** images of vacuum‐dried PPy nanoparticles were performed using a Carl Zeiss Ultra 55 field emission scanning electron microscope at a primary electron energy of 3.0 keV. The samples were adhered to conductive carbon tape and sputter‐coated with a 12 nm layer of Pd:Pt (20:80) before imaging.

Images for size analysis of PPy nanoparticles and DLP‐printed structures were obtained from a JEOL JSM‐7610F field emission scanning electron microscope equipped with a eucentric, 5‐axis motorized stage and three different detectors: semi‐in‐lens (SEI), lower off‐axis (LEI), and large‐angle backscatter (LABE) detectors. Imaging was conducted with a 1 kV acceleration voltage and a working distance of 6 mm at angles from 0° to 30°. The sample holders used were 12.5 mm in diameter, and vacuum‐dried samples were mounted using double‐sided adhesive carbon tape. Particle size analysis was conducted using ImageJ 1.54 g.

**Optical microscopy** was performed on an Olympus BX51 optical microscope equipped with Olympus objectives for 5× and 50× magnification. Bright‐field images were taken and processed using Stream Essentials software.

**DLS** to obtain particle size and distribution was done using a Malvern Panalytical Ultra ZSU5700 Zetasizer. Samples were prepared by suspending PPy in ultrapure water (0.05 mg/mL) and sonicated for 1 h. Measurements were performed at a backscattering angle of 173° using a quartz cuvette at 25 °C. After an equilibration time of 5 min, each sample was measured in triplicate, and the average results were reported as intensity‐weighted average hydrodynamic diameter.

**Profilometry** of DLP‐printed single layers for the calculation of conductivity was performed using a Bruker DektakXT tactile surface profilometer.

**CAD fidelity** in 3D‐printed structures was measured using a digital caliper, comparing the CAD of the printed object with the obtained structure.

**Conductivity measurements** were performed by measuring current–voltage (*I*/*V*) curves from −1.0 to +1.0 V in 51 steps at ambient conditions using a Keithley 2400 Source Meter controlled by a custom‐made Python script. The electrical conductivity was then calculated with respect to the geometry of the structure and the electrode setup (Section S6). Measurement of neat PPy pellets and 3D‐printed cubes was performed using the two‐point probe method. Herein, the structure was placed between two gold‐coated glass slides using a custom‐made vice and connected via gold‐coated clamps. For the measurements of printed single layers, custom‐made four‐line gold electrodes with an equal line spacing of 500 µm and an inner line width of 250 µm were used. The printed single layers were compressed onto the gold electrodes using a custom‐made vise, contacted by two or four tungsten needles, and characterized using either the two‐point probe or four‐point probe configuration.

**Electrical impedance measurements** were conducted on DLP‐printed single layers using an impedance analyzer by Zurich Instruments (MFIA 5 MHz) in the two‐contact mode. The software LabOne (V26.01.1.6, Zurich Instruments) was used to interface with the device. Impedance frequency sweeps were conducted in the range of 1 Hz–1 MHz (100 datapoints) using a perturbation potential of 10 mV at the “slow‐settling” settings provided by the software.

**Thermogravimetric analysis** was performed on a Mettler Toledo TGA 2 instrument in the temperature range between 30 °C and 600 °C with a heating rate of 10 °C/min under a nitrogen atmosphere. For this purpose, cubic samples (1.0 × 1.0 × 1.0 mm) were printed and dried overnight in a vacuum oven to remove residual solvents and moisture eventually present in the samples.

## Funding

This study was supported by Deutsche Forschungsgemeinschaft (GRK2948, EXC‐2082/2‐390761711, INST 35/1503‐1 FUGG).

## Conflicts of Interest

The authors declare no conflicts of interest.

## Supporting information

Supplementary Material

## Data Availability

The data that support the findings of this study are available from the corresponding author upon reasonable request.
