# Supplementary material for: 3D DLP Fabrication of Electrically Conductive Polypyrrole Nanocomposites
Source: Small Sci. 2026 Oct 7;6(10):e70422. doi: 10.1002/smsc.70422 (PMC13646484; doi:10.1002/smsc.70422)
Supplement: Supplementary file 1 [file SMSC-6-e70422-s001.pdf]

Supporting Information

3D DLP Fabrication of Electrically Conductive Polypyrrole Nanocomposites

Silas C. Eiden[a], Robert F. Sojka[a], Christian Delavier[a], Sebastian Grieger[b], Jana Zaumseil[b]
Eva Blasco*[a]

Table of Contents

1. Synthesis of PPy Nanoparticles 2
2. Raman Spectroscopic Data of PPy Nanoparticles 4
3. FT-IR Spectroscopic Data of PPy Nanoparticles 5
4. UV/Vis Spectroscopic Data of Aqueous PPy Nanoparticle Dispersions 6
5. Morphology and Particle Size Analysis of PPy Nanoparticles 8
6. Methodology for Conductivity Measurements 10
7. 3D Printing Methodology 14
8. Raman Spectroscopic Data of PPy-Fe-SDS Nanocomposites 18
9. FT-IR Spectroscopic Data of PPy-Fe-SDS Nanocomposites 20
10. Electrical Conductivities of Nanocomposite Single Layers 22
11. Electrical Impedance Spectroscopy of Nanocomposite Single Layers 23
12. Comparison with the State of the Art 24
13. Optical Microscopy Images of PPy-Fe-SDS Nanocomposites 24
14. SEM Images of Printed Nanocomposite 25
15. Thermogravimetric Analysis Data 26
16. Conductivities of Printed Nanocomposite Structures 26
17. Printability 27
18. References 28

1. Synthesis of PPy Nanoparticles

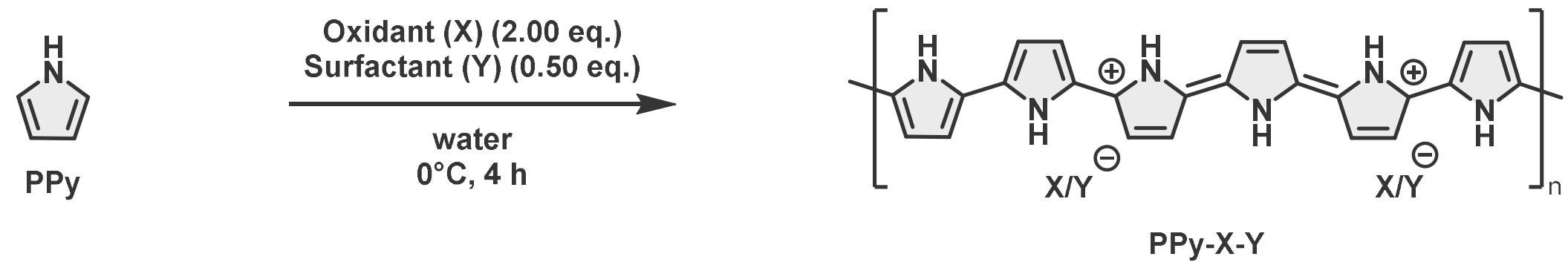

Scheme S1: Synthesis of doped PPy nanoparticles by chemical oxidative polymerization using 2.00 eq. of oxidant (either $FeCl_3$ **(Fe)** or **APS**) and 0.50 eq. of surfactant (either **SDS** or **DBSA**) in water at reduced temperature of 0 °C.

Following a modified literature procedure, pyrrole (336 mg, 5.00 mmol, 1.00 eq.) and the surfactant (Y, either SDS or DBSA) (2.50 mmol, 0.50 eq.) were dissolved in ultrapure water (10.0 mL) and stirred at 0 ºC for 10 min.[1] The oxidant (X, either $FeCl_3$ (Fe) or APS) (10.0 mmol, 2.00 eq.) was dissolved separately in ultrapure water (4.00 mL), cooled to 0 ºC using an ice bath and added dropwise to the reaction mixture. The resulting black suspension was stirred at 0 ºC for 4 h and subsequently quenched using MeOH (3.00 mL). The crude product was filtered off, washed with water (2 × 10.0 mL), MeOH (2 × 10.0 mL) and acetone (2 × 10.0 mL), then dried under reduced pressure (10^{-1} mbar, 40 ºC) for 16 h. Finally, the obtained black powder **PPy-X-Y** was finely ground and stored under nitrogen atmosphere at –19 ºC.

Table S1: Outcome of the synthesis of PPy nanoparticles using different combinations of oxidant (X) and surfactant (Y).

Product (PPy-X-Y)	Oxidant (X)	Surfactant (Y)	Outcome [mg][(a)]
PPy-Fe-SDS	$FeCl_3$	SDS	685
PPy-Fe-DBSA	$FeCl_3$	DBSA	820
PPy-APS-SDS	APS	SDS	317
PPy-APS-DBSA	APS	DBSA	448

(a) Product outcome was averaged over four repeated reactions.

PPy-Fe-SDS

UV/Vis (H_2O): λ_{max} = 484 nm.

IR (FT-ATR) (KBr pellet): $\tilde{\nu}$ = 3420 (m), 2919 (w), 2850 (w), 1540 (m), 1455 (m), 1303 (m), 1168 (s), 1087 (w), 1040 (s), 963 (m), 892 (s), 860 (s), 790 (m), 667 (m), 617 (s) cm^{-1}.

Raman: $\tilde{\nu}$ = 1575 (s), 1379 (m), 1347 (m), 1231 (m), 1079 (m), 1056 (m), 970 (m), 931 (m), 679 (w), 615 (w) cm^{-1}.

PPy-Fe-DBSA

UV/Vis (H_2O): λ_{max} = 224, 478 nm.

IR (FT-ATR) (KBr pellet): $\tilde{\nu}$ = 2922 (w), 2852 (w), 1542 (m), 1453 (m), 1300 (m), 1165 (s), 1089 (m), 1037 (s), 964 (m), 890 (s), 858 (s), 667 (m) cm^{-1}.

Raman: $\tilde{\nu}$ = 1575 (s), 1385 (m), 1336 (m), 1253 (m), 1079 (m), 1052 (m), 977 (m), 932 (m), 681 (w), 619 (w) cm^{-1}.

PPy-APS-SDS

UV/Vis (H_2O): λ_{max} = 496 nm.

IR (FT-ATR) (KBr pellet): $\tilde{\nu}$ = 3423 (w), 2919 (w), 2850 (w), 1708 (w), 1546 (m), 1456 (m), 1303 (m), 1168 (s), 1092 (m), 1040 (s), 963 (m), 890 (s), 859 (s), 788 (m), 669 (m) cm^{-1}.

Raman: $\tilde{\nu}$ = 1728 (w), 1632 (m), 1594 (s), 1500 (m), 1420 (m), 1383 (m), 1355 (m), 1308 (m), 1261 (m), 1082 (m), 1055 (m), 985 (m), 931 (m), 695 (w) cm^{-1}.

PPy-APS-DBSA

UV/Vis (H_2O): λ_{max} = 224 nm.

IR (FT-ATR) (KBr pellet): $\tilde{\nu}$ = 3422 (w), 2922 (w), 2851 (w), 1712 (w), 1556 (m), 1456 (m), 1407 (m), 1320 (m), 1174 (s), 1036 (s), 966 (m), 900 (s), 669 (m) cm^{-1}.

Raman: $\tilde{\nu}$ = 1567 (s), 1336 (s) cm^{-1}.

2. Raman Spectroscopic Data of PPy Nanoparticles

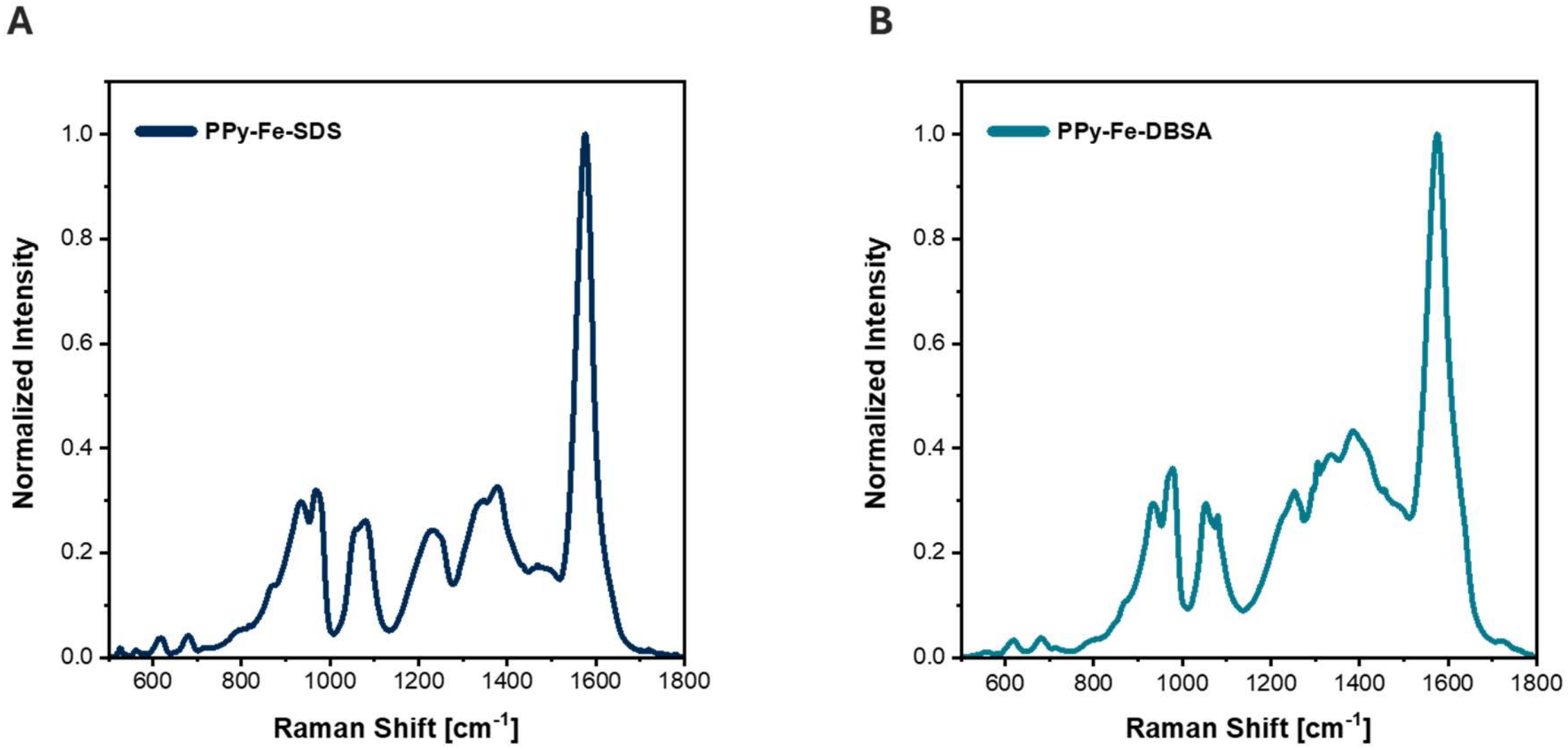

Figure S1: Normalized Raman spectra of A) **PPy-Fe-SDS** and B) **PPy-Fe-DBSA.**

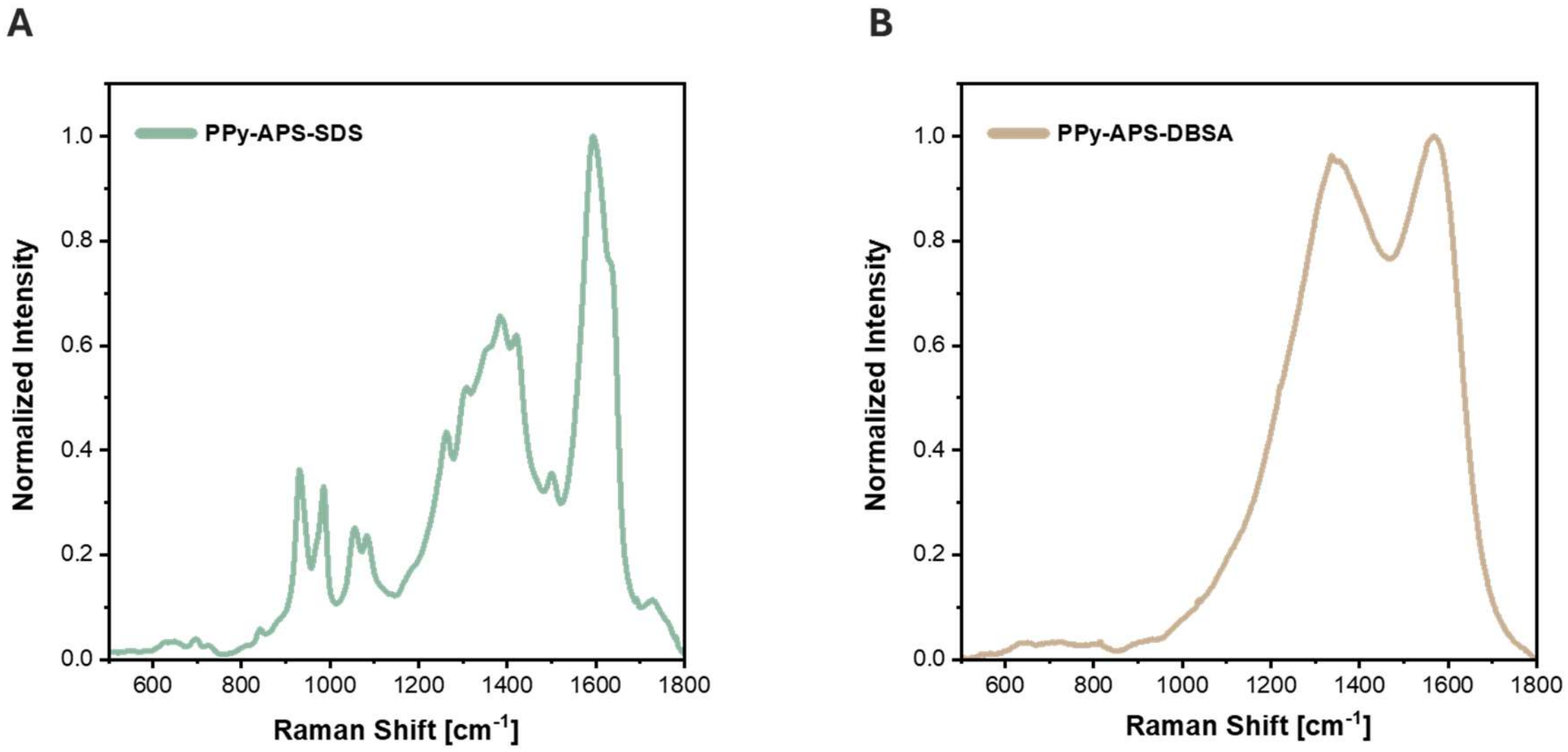

Figure S2: Normalized Raman spectra of A) **PPy-APS-SDS** and B) **PPy-APS-DBSA**.

3. FT-IR Spectroscopic Data of PPy Nanoparticles

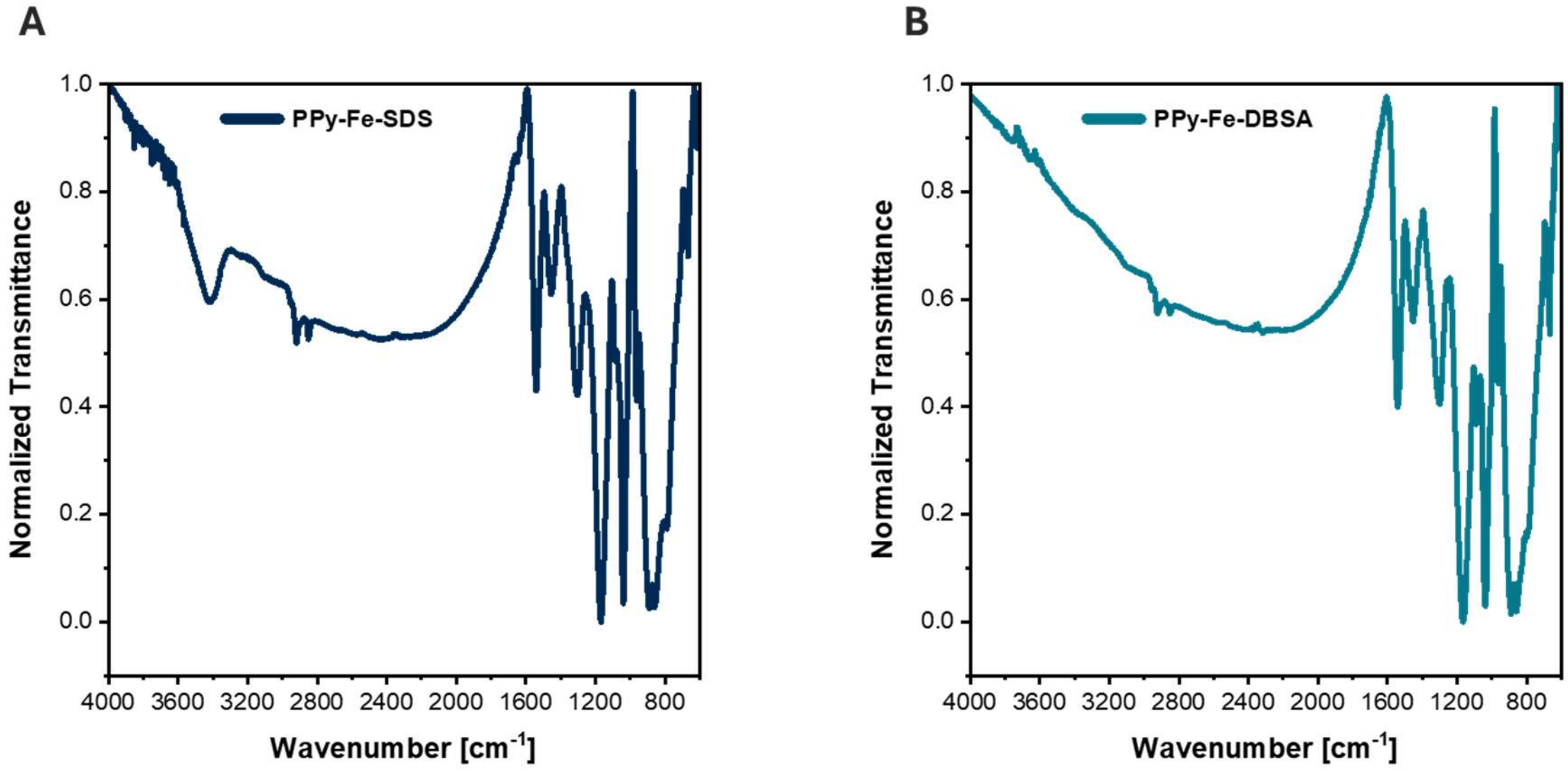

Figure S3: FT-IR spectra of PPy/KBr pellets of A) **PPy-Fe-SDS** and B) **PPy-Fe-DBSA**.

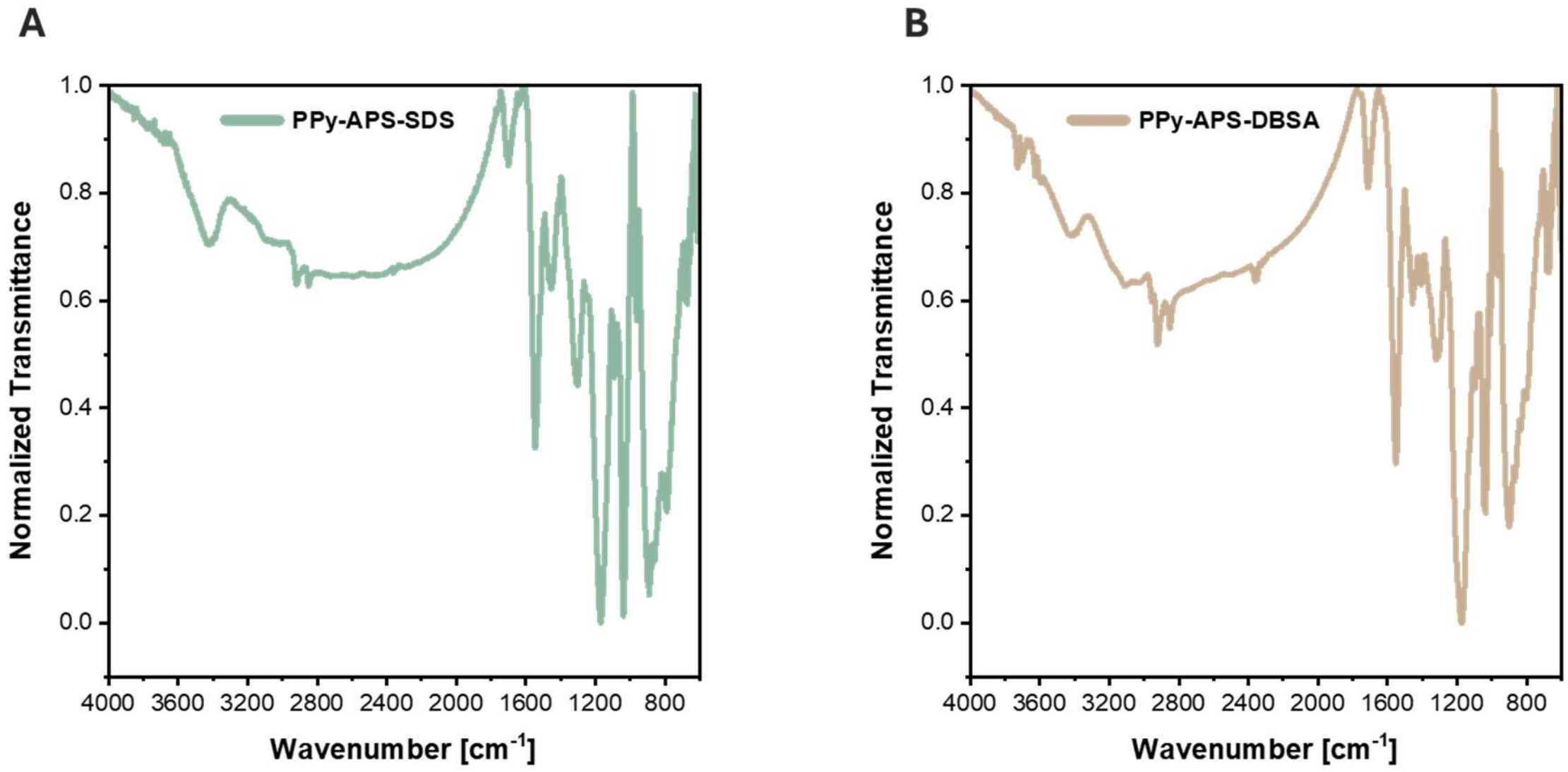

Figure S4: FT-IR spectra of PPy/KBr pellets of A) **PPy-APS-SDS** and B) **PPy-APS-DBSA**.

4. UV/Vis Spectroscopic Data of Aqueous PPy Nanoparticle Dispersions

Aqueous PPy Nanoparticle Dispersions

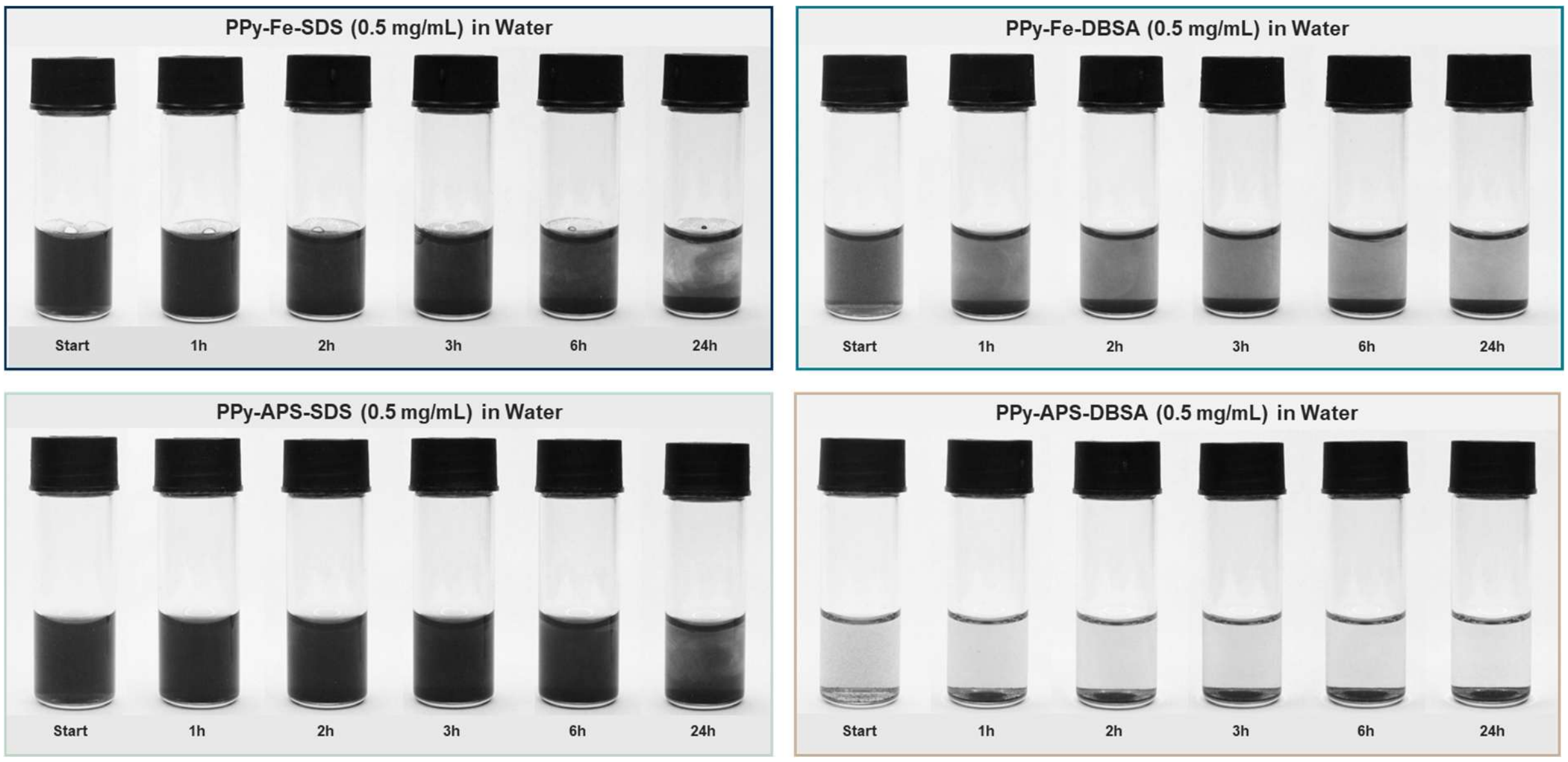

Figure S5: Aqueous dispersions (0.5 mg/mL) of **PPy-Fe-SDS**, **PPy-Fe-DBSA**, **PPy-APS-SDS** and **PPy-APS-DBSA** taken directly after ultrasonication for 1 h and again after progressively longer rest periods up to 24 h.

UV/Vis Spectroscopic Data

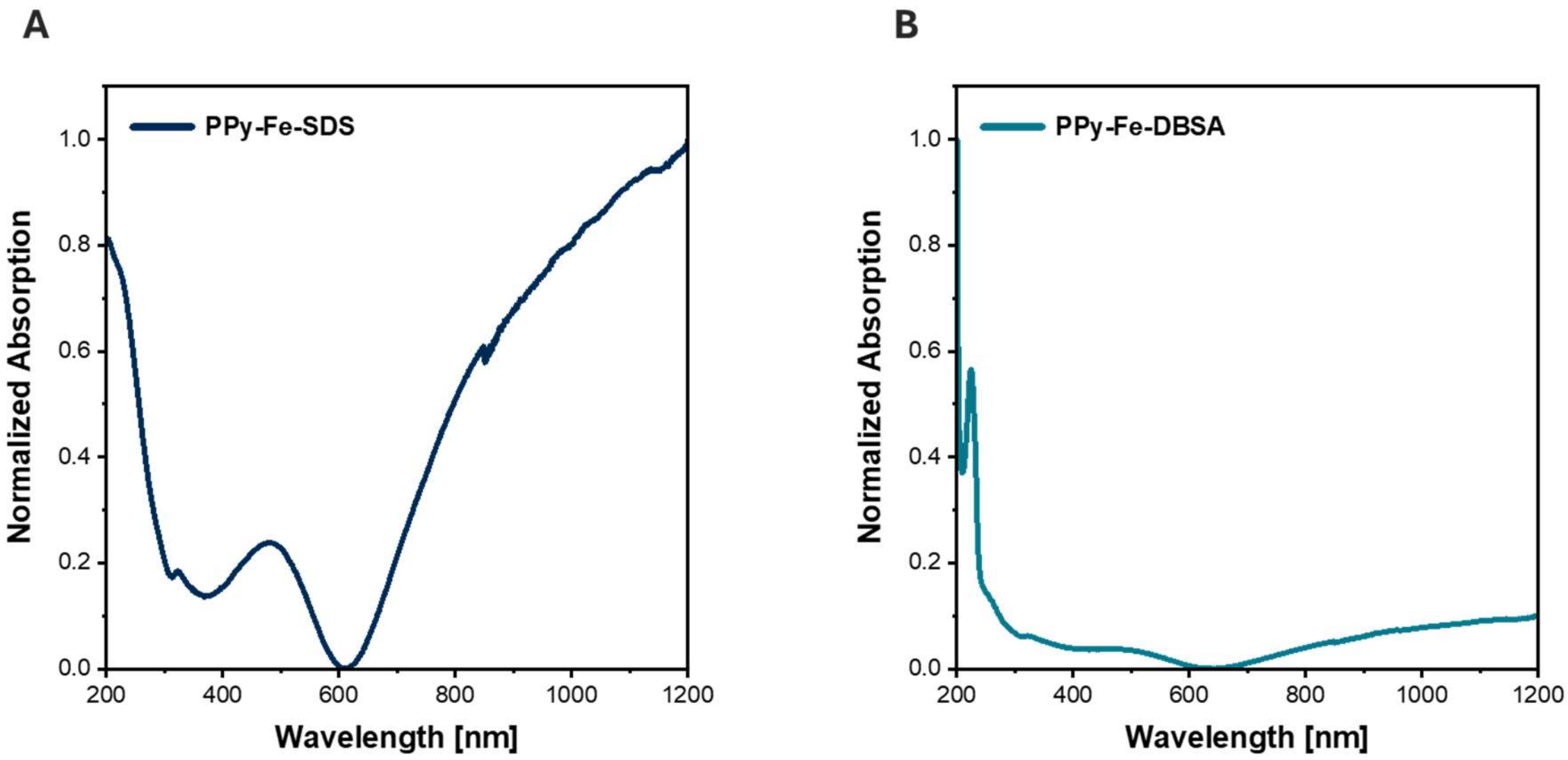

Figure S6: Normalized UV/Vis spectra of aqueous dispersions (0.05 mg/mL) of A) **PPy-Fe-SDS** and B) **PPy-Fe-DBSA**.

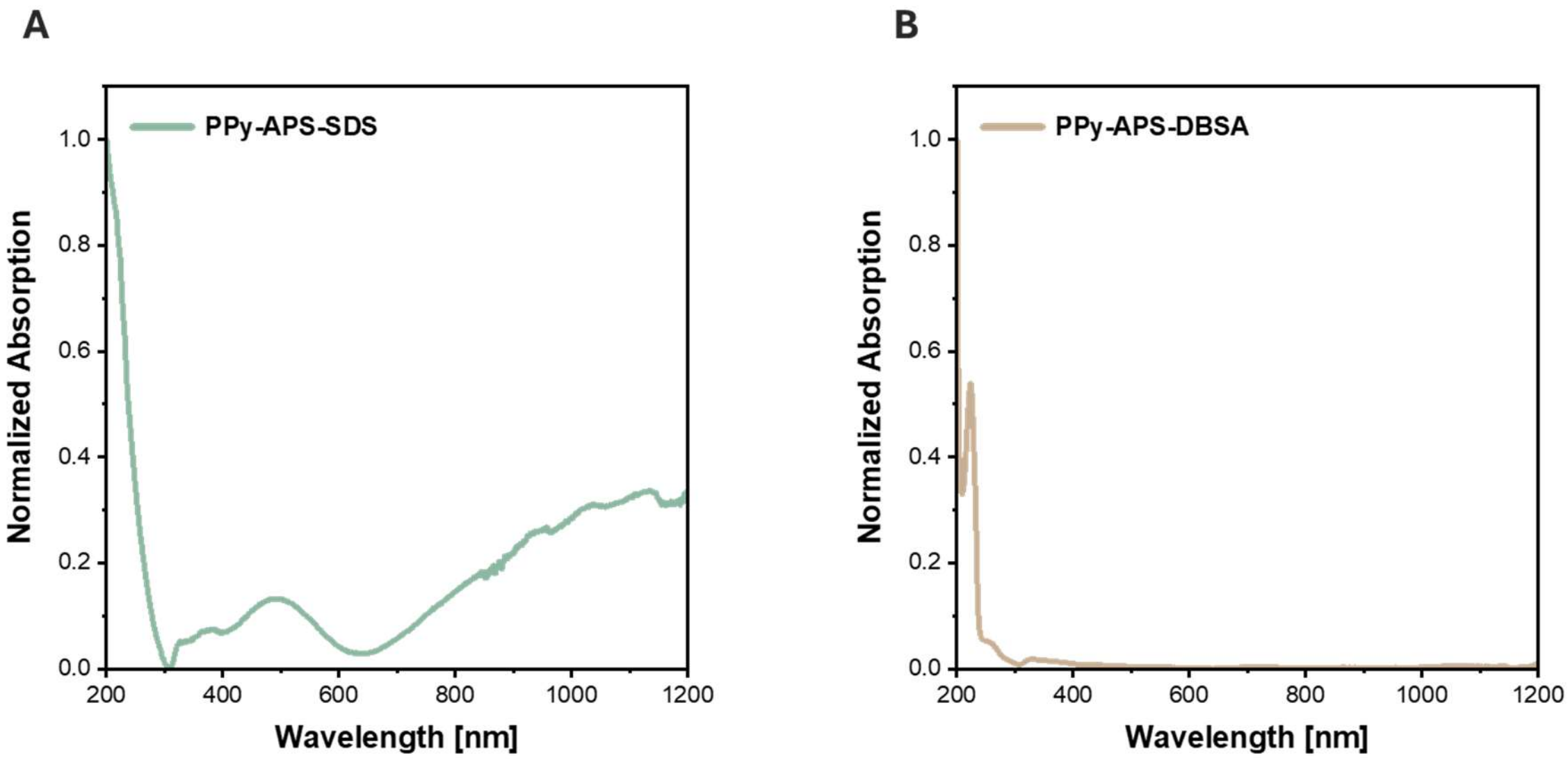

Figure S7: Normalized UV/Vis spectra of aqueous dispersions (0.05 mg/mL) of A) **PPy-APS-SDS** and B) **PPy-APS-DBSA**.

5. Morphology and Particle Size Analysis of PPy Nanoparticles

SEM of Bulk PPy Nanoparticles

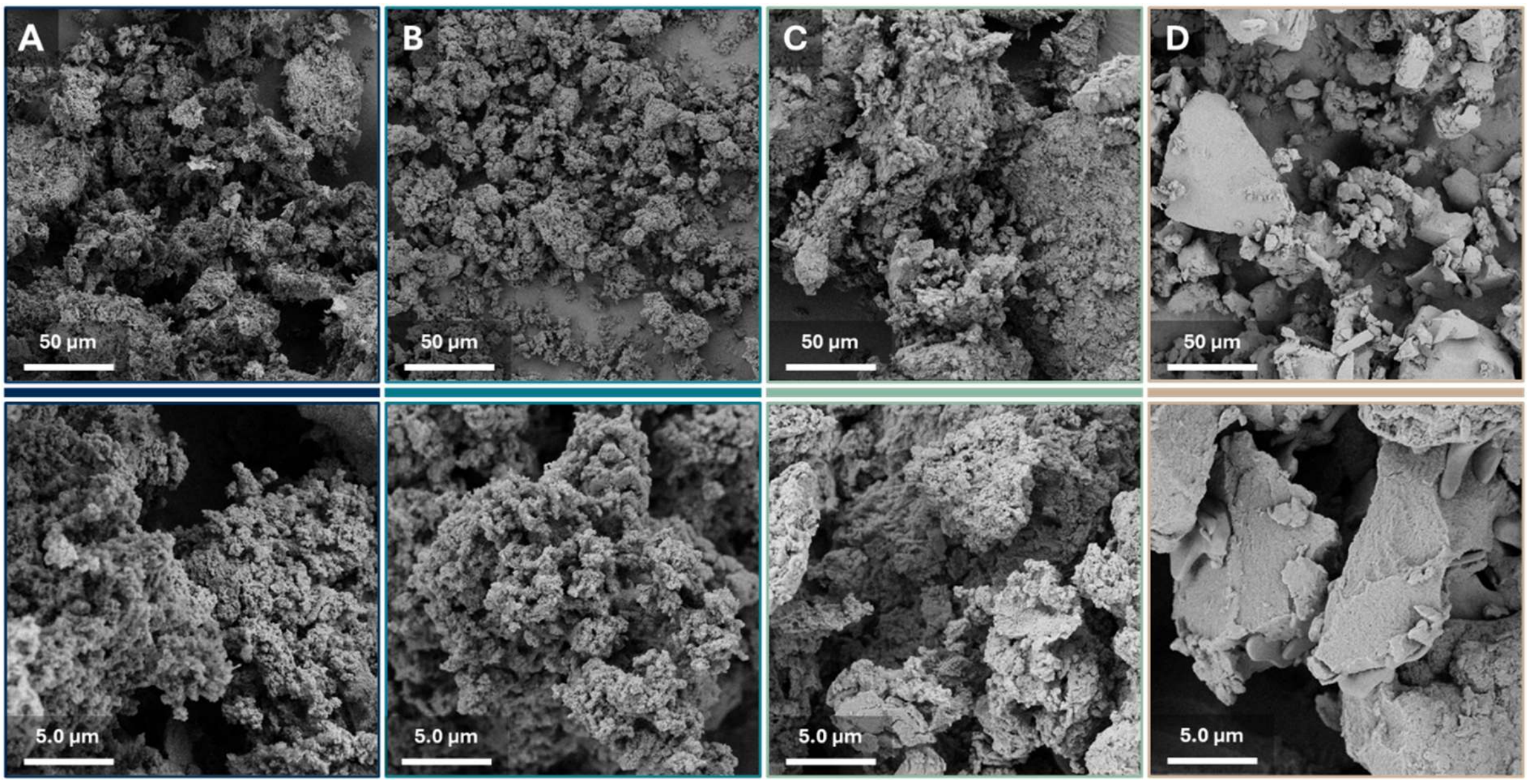

Figure S8: SEM images of sputter-coated bulk PPy nanoparticles. A) **PPy-Fe-SDS**. B) **PPy-Fe-DBSA**. C) **PPy-APS-SDS**. D) **PPy-APS-DBSA.**

Surfactant Stabilized Aqueous Dispersions

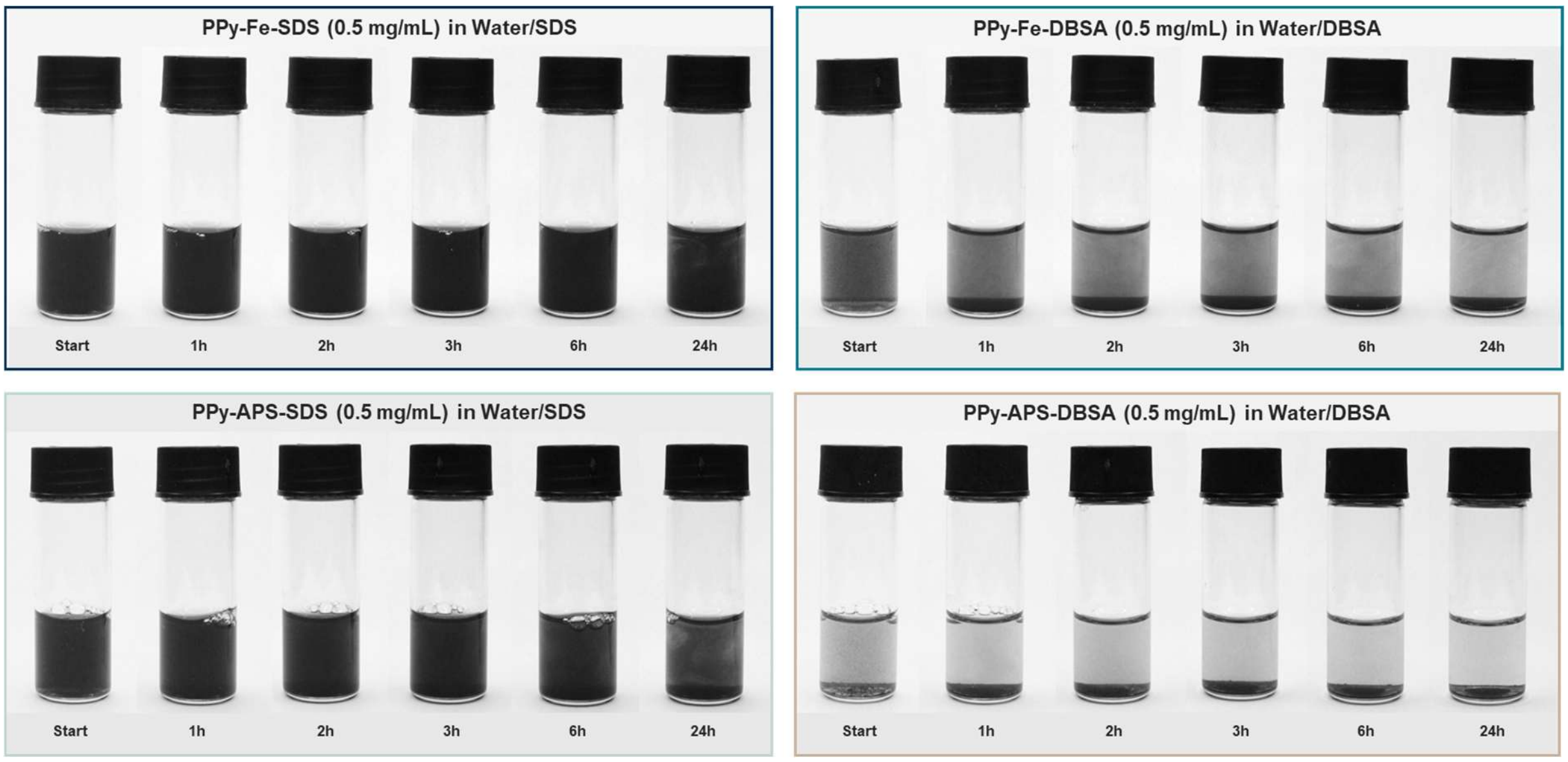

Figure S9: Aqueous dispersions (0.5 mg/mL) of **PPy-Fe-SDS**, **PPy-Fe-DBSA**, **PPy-APS-SDS** and **PPy-APS-DBSA** taken directly after ultrasonication for 1 h and again after progressively longer rest periods up to 24 h. For stabilization of the dispersions, 5.0 mg/mL of the respective surfactant SDS or DBSA was added to the mixture prior to ultrasonication.

SEM of PPy Nanoparticles from Surfactant Stabilized Aqueous Dispersions

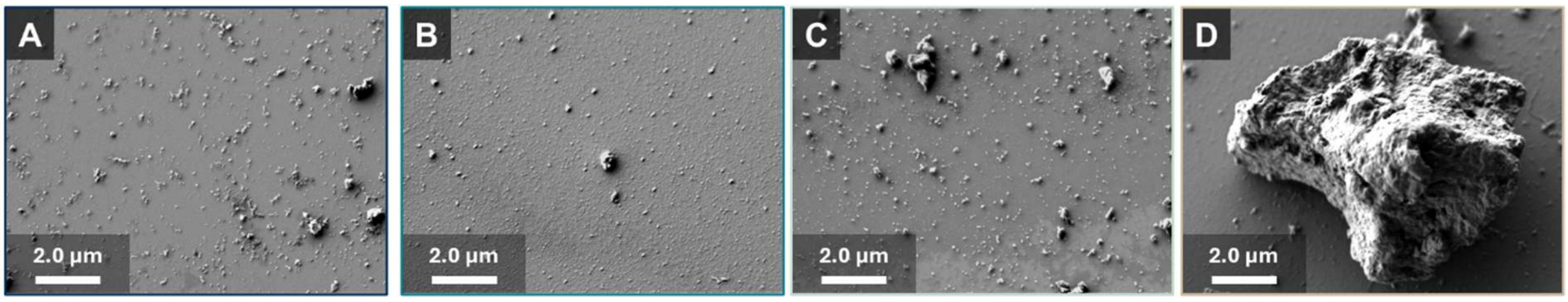

Figure S10: SEM images of single PPy nanoparticles films deposited from surfactant (5.0 mg/mL) stabilized aqueous dispersions (0.5 mg/mL) of (A) **PPy-Fe-SDS,** (B) **PPy-Fe-DBSA** and (C) **PPy-APS-SDS** allowed distinct particle size analysis. D) Strong agglomeration of **PPy-APS-DBSA** revealed large, deposited particles.

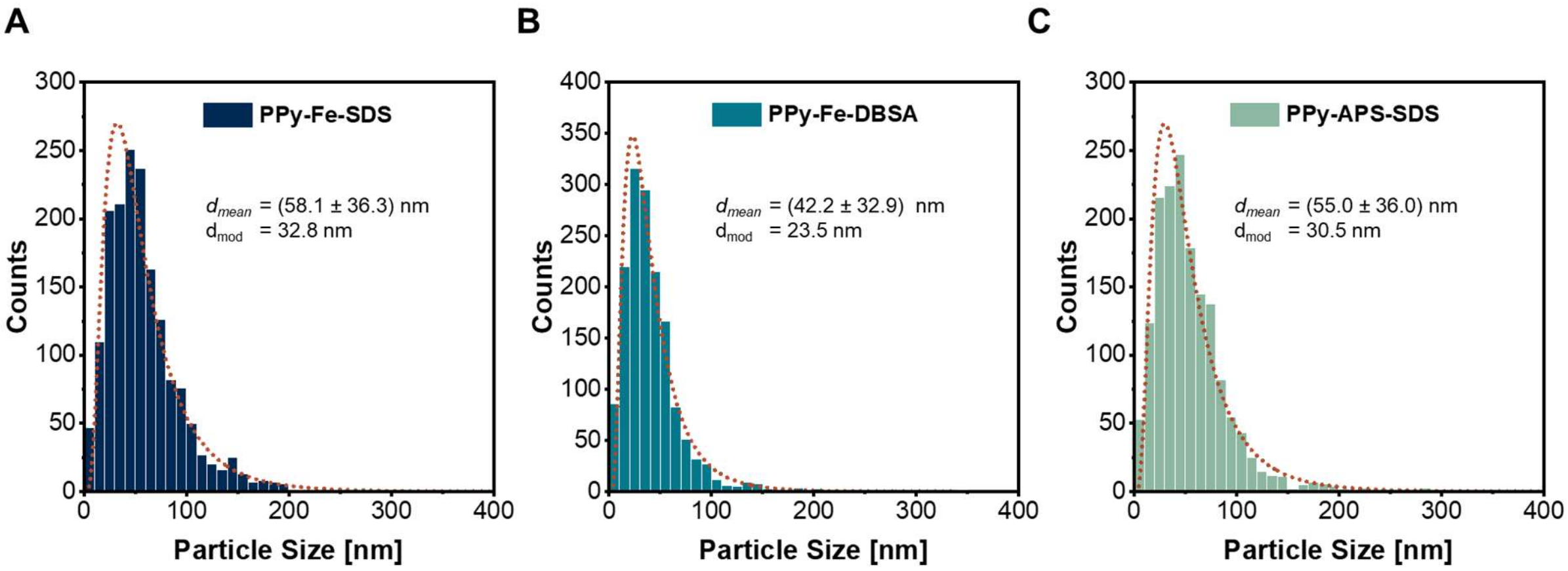

Figure S11: Particle size distribution of single PPy nanoparticles films deposited from surfactant (5.0 mg/mL) stabilized aqueous dispersions (0.5 mg/mL) of (A) **PPy-Fe-SDS**, (B) **PPy-Fe-DBSA** and (C) **PPy-APS-SDS**.

DLS Measurements

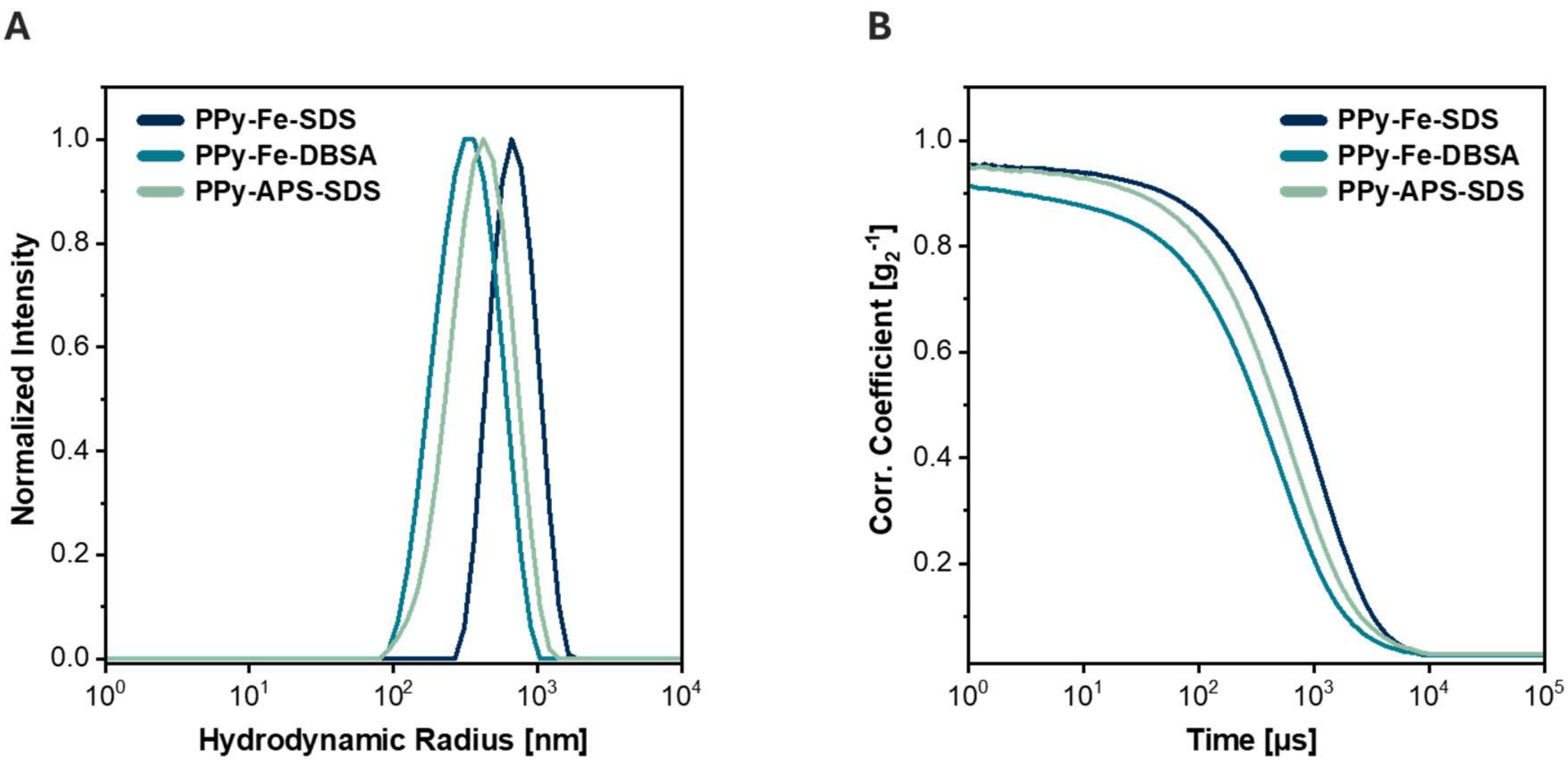

Figure S12: A) Dynamic light scattering measurements of dispersions of PPy nanoparticles (0.05 mg/mL) in water with the respective surfactant SDS (5.0 mg/mL) for **PPy-Fe-SDS** and **PPy-APS-SDS** and DBSA (5.0 mg/mL) for **PPy-Fe-DBSA**. For **PPy-APS-DBSA** no stable dispersions for DLS measurements could be obtained. B) Correlation Coefficient for each measurement.

6. Methodology for Conductivity Measurements

For the measurement of the electronic conductivity of all structures, current-voltage (I/V) curves from –1.0 to +1.0 V were measured in 51 steps at ambient conditions using a *Keithley 2400 Source Meter* controlled by a custom-made *Python* script. Plotting the measured currents I against the corresponding voltages V follows a linear relationship, whose slope represents the conductance G in accordance with Ohm's law:

$$G = \frac{dI}{dV}$$

Each sample was measured five times under identical conditions to obtain the mean conductance $\bar{G}$. The conductivity σ was then calculated as:

$$\sigma = \bar{G}\frac{L}{A}$$

Herein, L is the length of the current path and A is the cross-sectional area of the sample. The error of the conductivity $\Delta\sigma$ was determined using Gaussian error propagation:

$$\Delta\sigma = \sqrt{\left(\frac{\partial\sigma}{\partial\bar{G}}\Delta\bar{G}\right)^2} = \Delta\bar{G}\frac{L}{A}$$

Herein, $\Delta\bar{G}$ is the standard deviation from the conductance measurements. As three samples were printed with the same ink formulation, an average conductivity $\bar{\sigma}$ was calculated from the conductivity of the individual samples. The corresponding error of the average conductivity $\Delta\bar{\sigma}$ equals the standard deviation.

Two-Point Probe of PPy Pellets and 3D Printed Cubes

Conductivity of neat PPy pellets and 3D-printed cubes was performed using the two-point probe method. Herein, the structure was placed between two gold-coated glass slides (thermally evaporated layer of 100 nm gold on 2 nm chromium using a custom-made vise and connected *via* gold-coated clamps (**Figure S13**).

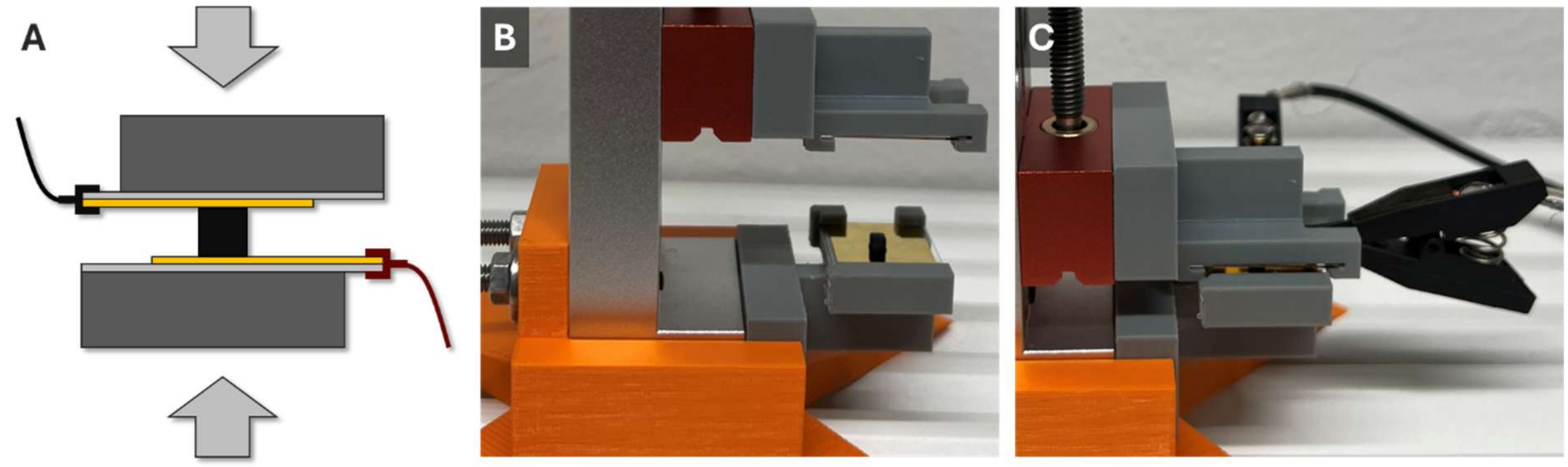

Figure S13: Two-point probe setup for the measurement of neat PPy pellets and DLP-printed cubes. A) Schematic representation of the structure fixated between two gold-coated glass slides, which are contacted by gold-coated clamps. B) Image of the open custom-made vise with a structure on top the bottom electrode. C) Image of the closed vise with the gold electrodes contacted from different sides to avoid bypassing.

Geometric factors for neat PPy pellets:

$$\sigma = \bar{G}\frac{L}{A} = \bar{G}\frac{t}{\pi(d/2)^2}$$

Herein, t is the thickness of the pellet and d is the respective diameter, both determined using a digital caliper.

Geometric factors for printed cubes:

$$\sigma = \bar{G}\frac{L}{A} = \bar{G}\frac{t}{lw}$$

Herein, t is the bulk thickness of the cube, l is the length and w is the width. Dimensions were taken directly from the CAD file and cross-checked after the printing using a digital caliper.

Four-Point and Two-Point Probe of Printed Single Layers

For the measurements of printed single layers, a custom-made four-line gold electrode with an equal line spacing of 500 µm and inner line width of 250 µm was used (**Figure S3**). The printed single layers were compressed onto the gold electrodes using a custom-made vise, contacted by two or four tungsten needles and characterized using either the two-point probe or four-point probe configuration.

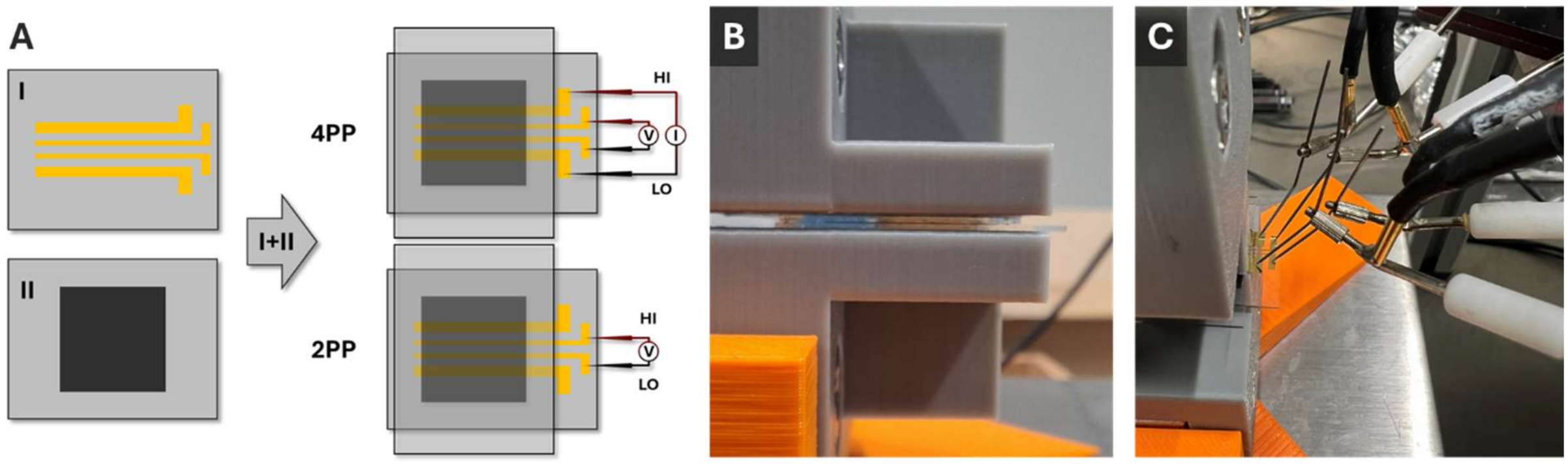

Figure S14: Setup for the measurement of DLP-printed single layers allowing for two-point and four-point probe measurements. A) Schematic representation of the four gold-line electrodes thermally evaporated onto glass (I) and the single layer printed onto a separate glass slide (II). By compressing both samples with electrodes and single layer facing the inside (I+II), four-point and two-point probe measurements can be done. The orthogonal alignment is considered in the calculation of conductivity. B) Image of the closed custom-made vise with the compressed sandwich of samples ensuring equal contact. C) Image of the closed vise with pads of each gold line accessible for contact needles.

Geometric factors for printed single layers:

$$\sigma = \bar{G}\frac{L}{A} = \bar{G}\frac{L}{tw}$$

Herein, t is the thickness of the single layer, w the width of the channel, limited by the width of the single layer, and L is the spacing of the electrodes. For both measurement methods, L equals the distance of the inner gold lines (L = 500 µm).

Electrical Impedance Spectroscopy of Printed Single Layers

Electrical impedance spectroscopy was performed on single layers DLP printed directly onto gold electrodes. Silver paint was carefully applied to the uncovered bottom electrode as well as to the back and side of the circular gold electrode. Resistance from the silver to the gold side was measured to be in the range of 2-3 Ω. Subsequently, the printed single layer was sandwiched between both electrodes and contacted carefully (**Figure S15**). Temperature dependent measurements were conducted under a steady stream of dry nitrogen. Measurements at different levels of relative humidity were performed using a custom-made setup providing a steady stream of humidified nitrogen of desired relative humidity. The impedance data was validated using the Kramer-Kronig validation method[2] via the software *Lin-KK* (distributed by KIT, Germany).[3–5]

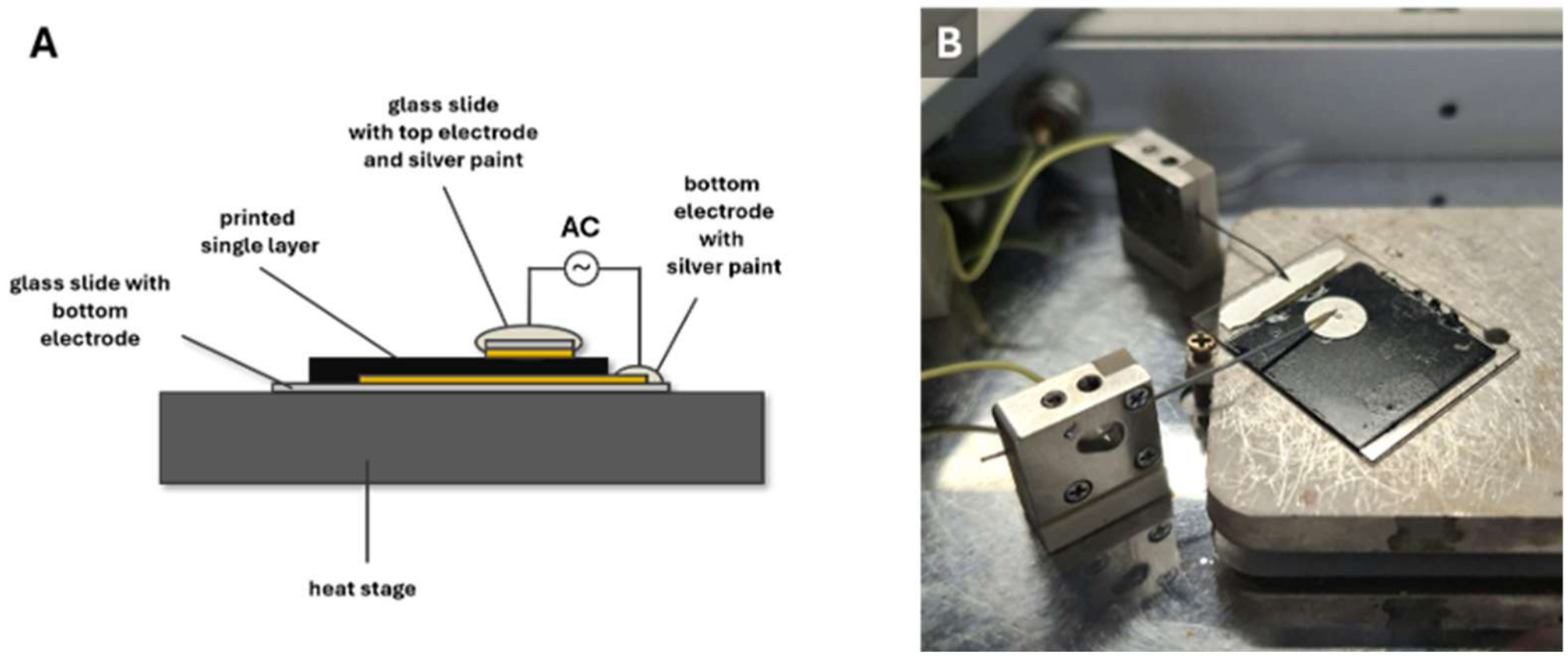

Figure S15: Experimental setup for electrical impedance measurements. A) Schematic depiction of the printed single layer, sandwiched between top and bottom electrode. B) Photograph of contacted **Sample B** on top of the heat stage.

The conductivity was determined using the following equation:

$$\sigma = \frac{L}{R \cdot A}$$

were σ denotes the conductivity, L denotes the thickness of polymer material and A denotes the electrode/polymer contact area. R denotes the bulk resistance observed in the impedance measurement.

7. 3D Printing Methodology

Ink Formulation

EGPEA (77.2 – 83.0 wt% relative to the total ink) and PEGDA700 (14.8 – 16.0 wt%) served as the base matrix of the formulation. The photoinitiator BAPO (1.0 wt%) and finely ground PPy (0 – 6.0 wt%) was incorporated into the base ink. Each formulation was sonicated for 1 h to achieve homogeneous dispersion of the filler. The resulting dispersions were directly used for material testing or printing to prevent agglomeration of filler particles.

Table S2: Overview of ink formulations containing **PPy filler**, monomer **EGPEA**, crosslinker **PEGDA700** and photoinitiator **BAPO**. All amounts are given in wt% in respect to the total amount of ink.

PPy Filler [wt%]	EGPEA [wt%]	PEGDA700 [wt%]	BAPO [wt%]
-	83.0	16.0	1.0
3.0	80.6	15.4	1.0
4.0	79.7	15.3	1.0
5.0	78.8	15.2	1.0
6.0	78.0	15.0	1.0

Ink Stability

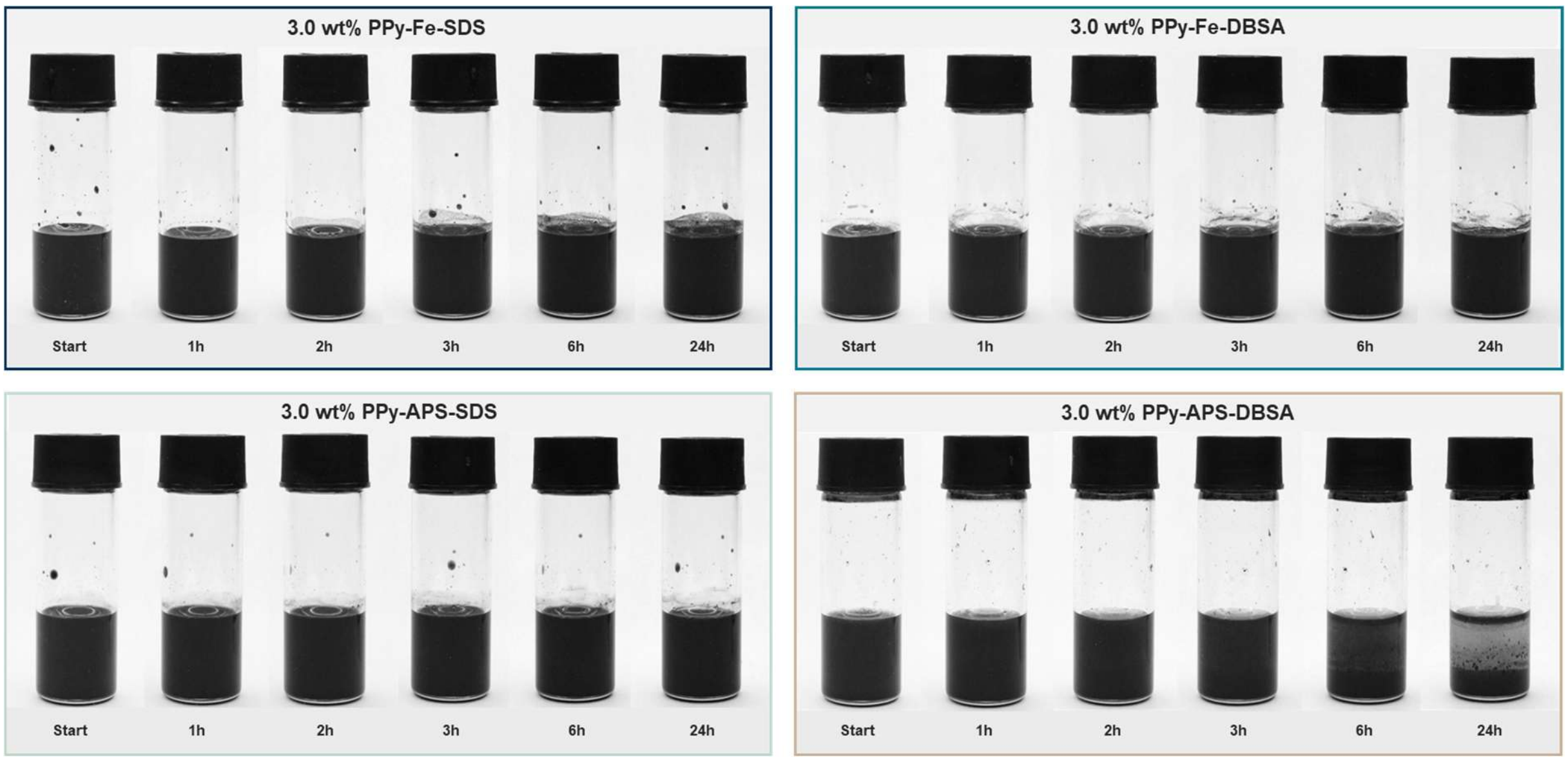

Figure S16: Inks containing 3.0 wt% of **PPy-Fe-SDS**, **PPy-Fe-DBSA**, **PPy-APS-SDS** and **PPy-APS-DBSA** directly after ultrasonication for 1 h and again after progressively longer rest periods up to 24 h.

DLP 3D Printing Setup

For DLP printing, an *Asiga MAX X27* (405 nm, 27 × 27 µm^2 pixel size) was used together with a custom aluminum build platform (2.5 × 2.5 cm^2) and a modified ink vat based on a *MonoPrinter* design. Printer compatibility was achieved through a self-designed FDM-printed adapter. The FEP foil (50 µm) of the vat was replaced before each session to ensure reproducibility. Structures were modeled in *Blender 4.0.1* and exported as *.stl* files. Printing parameters and file upload were handled via *Asiga Composer 2.0.4*.

Material Tests

For each ink batch, exposure conditions were first screened. A drop of ink on a microscope cover glass (22 × 22 mm, thickness: 170 ± 5 µm) was illuminated with a 2.0 mm light spot for defined times and intensities. After rinsing off uncured material with isopropanol and drying with N_2, the thickness of the cured layer was measured using a caliper. Plotting the resulting curing depth (C_d) against the natural logarithm of the applied light energy per area (E_0) yielded a linear relationship consistent with Jacob's working curve:

$$C_d = D_p \ln\left[\frac{E_0}{E_c}\right]$$

By performing a linear fit of the measured data, one can determine the minimal energy required for photopolymerization (E_c) and the penetration depth (D_p) for each material.

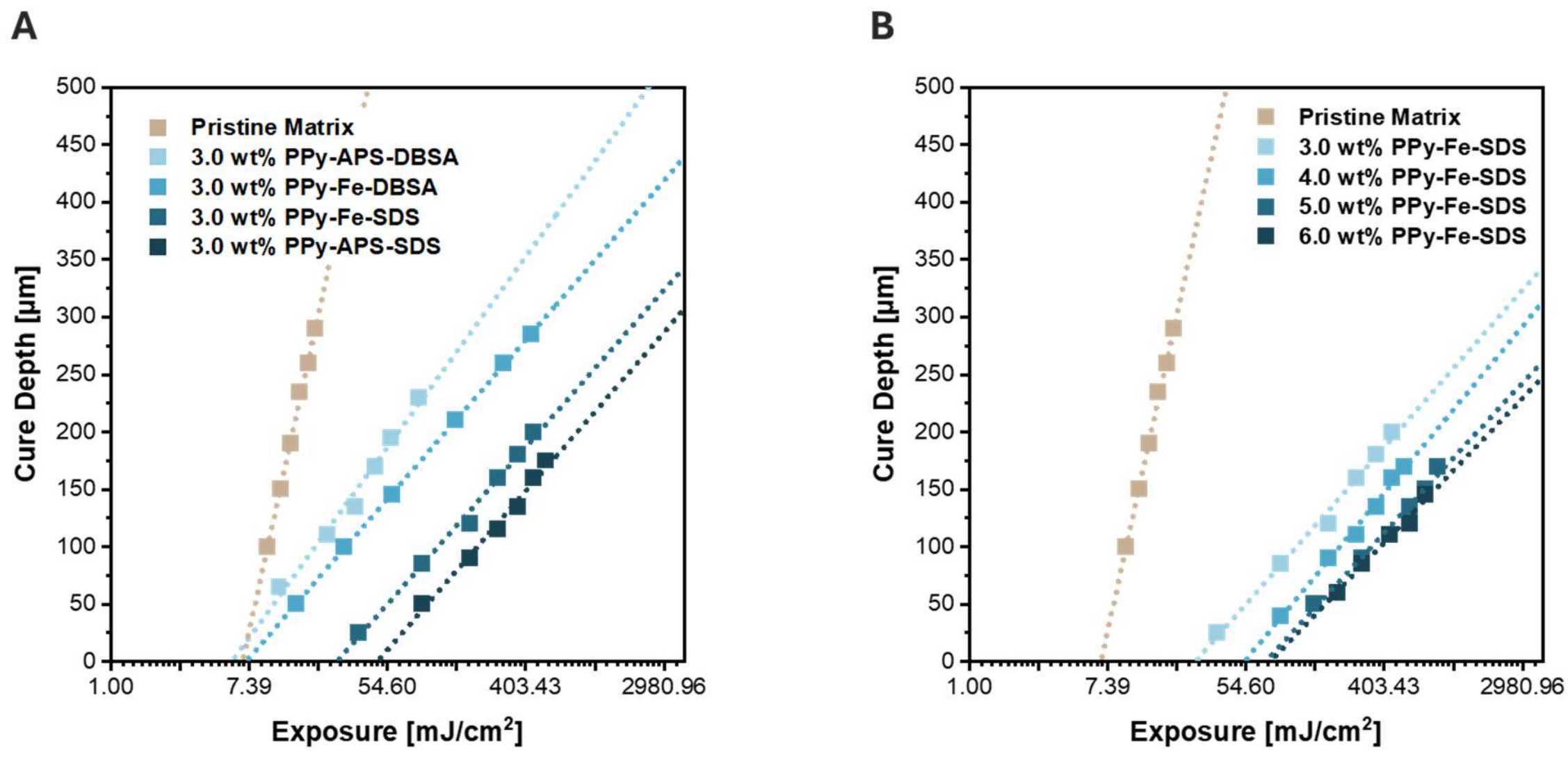

Figure S17: A) Jacobs working curves for filler screening using 3.0 wt% of **PPy-Fe-SDS**, **PPy-Fe-DBSA**, **PPy-APS-SDS** and **PPy-APS-DBSA**, respectively. B) Jacobs working curves for inks containing 0 – 6.0 wt% **PPy-Fe-SDS**.

Table S3 Overview of penetration depth (D_p) and critical curing energy (E_c) derived from the Jacobs working curves of respective ink formulations containing **PPy filler**, monomer **EGPEA**, crosslinker **PEGDA700** and photoinitiator **BAPO**.

PPy Filler	D_p [µm]	E_c [mJ/cm^2]
Pristine Matrix	275 ± 5.63	6.68 ± 1.02
3.0 wt% PPy-Fe-SDS	68.4 ± 3.16	26.2 ± 1.10
3.0 wt% PPy-Fe-DBSA	69.3 ± 0.52	7.06 ± 1.02
3.0 wt% PPy-APS-SDS	69.2 ± 4.19	47.0 ± 1.11
3.0 wt% PPy-APS-DBSA	83.0 ± 5.55	5.75 ± 1.13
3.0 wt% % PPy-Fe-SDS	68.4 ± 3.16	26.2 ± 1.10
4.0 wt% PPy-Fe-SDS	72.6 ± 3.54	53.3 ± 1.09
5.0 wt% PPy-Fe-SDS	65.3 ± 3.84	71.5 ± 1.11
6.0 wt% PPy-Fe-SDS	62.9 ± 4.65	77.5 ± 1.13

In theory, the exposure time for a given light intensity and target curing depth can be calculated directly from the working curve parameters. In practice, the measured curing-depth data were entered into *Asiga Composer 2.0.4*, which uses proprietary algorithms to determine the appropriate exposure time for the selected light intensity and layer height.

DLP 3D Printing on Silanized Glass Substrate

The silanized glass substrate was mounted on the build plate with the treated side facing away from the build platform and secured using double-sided adhesive tape (*tesa 64621-00000*). After attaching the build platform, z-axis calibration and build platform leveling were performed individually for each substrate. Before uploading the models, their xy-positions in *Asiga Composer* were aligned with the actual placement of the glass substrate within the printer's build area. After printing, structures were rinsed with isopropanol, dried with pressurized N_2, and the substrate was detached from the platform. Residual adhesive on the back side was removed with acetone.

DLP 3D Printing on Aluminum Build Platform

For the preparation, the aluminum build platform was cleaned with acetone and attached to the printer following z-axis calibration and build platform levelling prior to printing. Post-processing of the printed structures was performed by rinsing the structure with isopropanol. Excess solvent was dried using pressurized N_2 and the structure was removed from the build platform.

Overview of Printed Structures and Printing Settings

Rectangular single layers with an edge length 15.0 × 15.0 mm and a layer thickness of 50 µm were printed onto silanized glass slides (for printing settings see **Table S4**). Rectangular single layers (18.0 × 18.0 mm) with a thickness of 50 µm for electrical impedance spectroscopy were printed onto silanized glass slides comprising evaporated gold electrodes. Cubes for conductivity measurements with an edge length of 2.5 × 2.5 mm and different bulk thickness in measurement direction of 1.5 – 2.5 mm were printed directly onto the aluminum build platform. Cubes for thermogravimetric analysis (1.0 mm × 1.0 mm × 1.0 mm) were printed directly onto the custom-made aluminum build platform.

The following structures:

- sunflower (CAD: 15.0 mm × 15.0 mm × 1.30 mm),
- snowflake (CAD: 13.4 mm × 15.0 mm × 0.50 mm),
- chess piece (CAD: 7.55 × 7.55 × 11.25 mm)
- benchy structure (CAD: 15.1 × 7.81 × 12.0 mm)

were printed with a layer thickness of 50 µm and five consecutive burn-in layers (exposure time = 20 s each) directly onto the aluminum build platform.

Table S4: Optimized printing parameters for each ink formulation containing **PPy filler**, monomer **EGPEA**, crosslinker **PEGDA700** and photoinitiator **BAPO** regarding exposure time, light intensity and layer thickness used for all single-layer and multilayer structures.

PPy Filler	Exposure Time [s]	Intensity [mW/cm^2]	Layer Thickness [µm][(a)]
Pristine Matrix	4.9	2.0	50
3.0 wt% PPy-Fe-SDS	4.0	18	50
3.0 wt% PPy-Fe-DBSA	6.7	18	50
3.0 wt% PPy-APS-SDS	8.1	18	50
3.0 wt% PPy-Fe-SDS	4.0	18	50
4.0 wt% PPy-Fe-SDS	8.5	18	50
5.0 wt% PPy-Fe-SDS	30	29	50

(a) Given layer thickness was set in the printing settings, the actual thickness of the printed single layer required for conductivity calculation was obtained after the printing process using a profilometer.

8. Raman Spectroscopic Data of PPy-Fe-SDS Nanocomposites

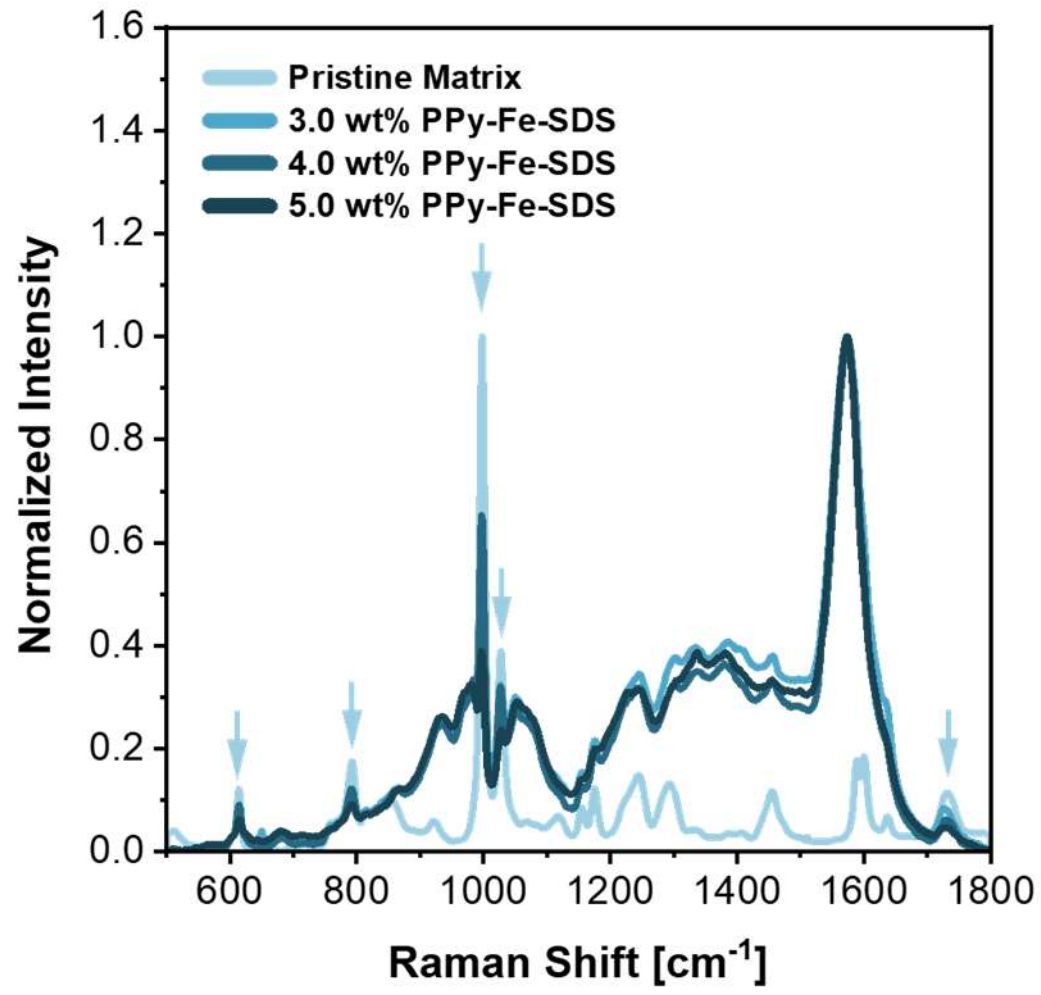

Figure S18: Stacked normalized Raman spectra of single layers DLP-printed with 0 – 5.0 wt% of **PPy-Fe-SDS** reveals decreasing signal intensity for matrix component **EGPEA** related signals and growing **PPy-Fe-SDS** content.

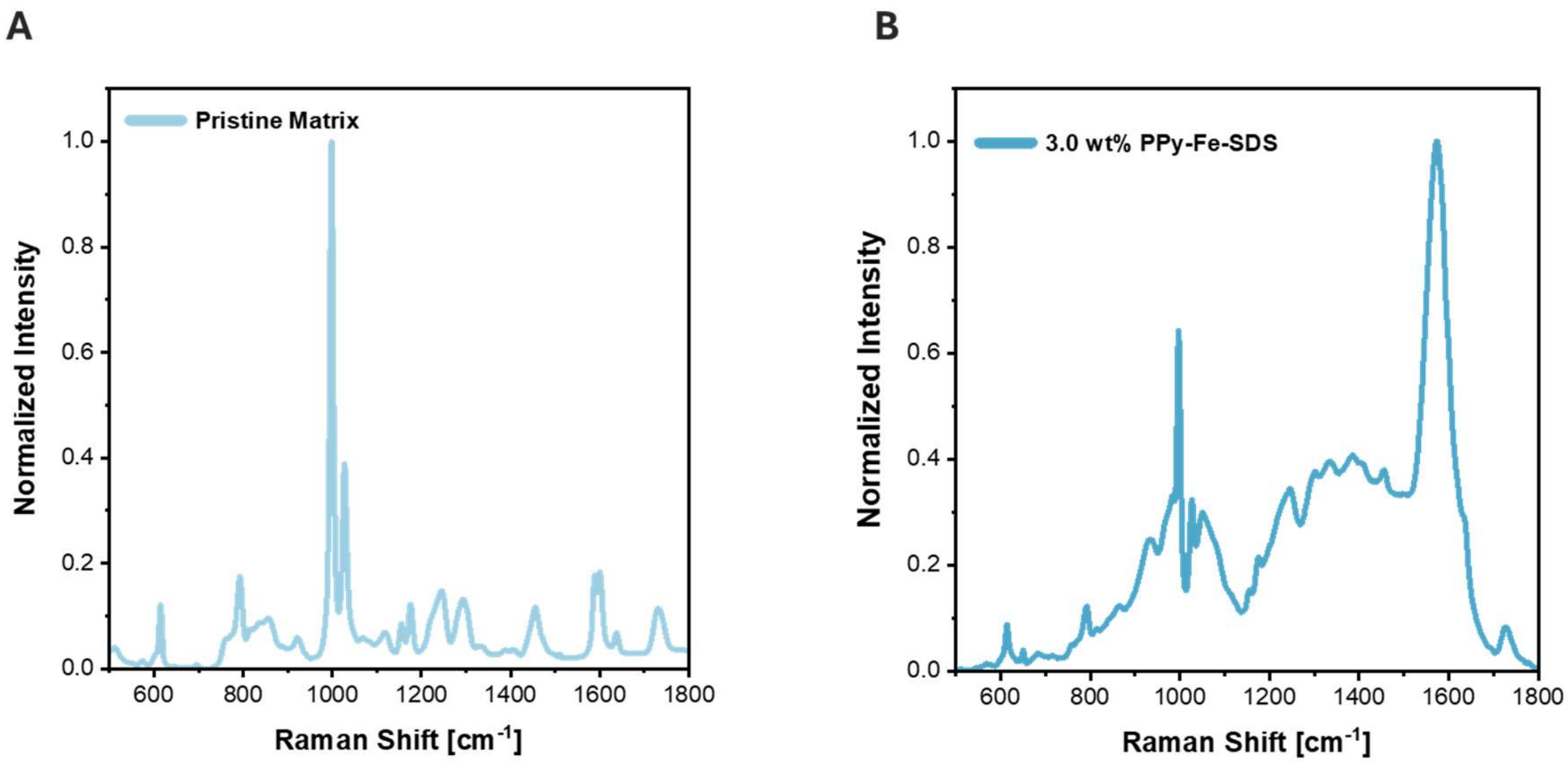

Figure S19: Normalized Raman spectra of A) the pristine matrix and B) single layer printed with inks containing 3.0 wt% **PPy-Fe-SDS**.

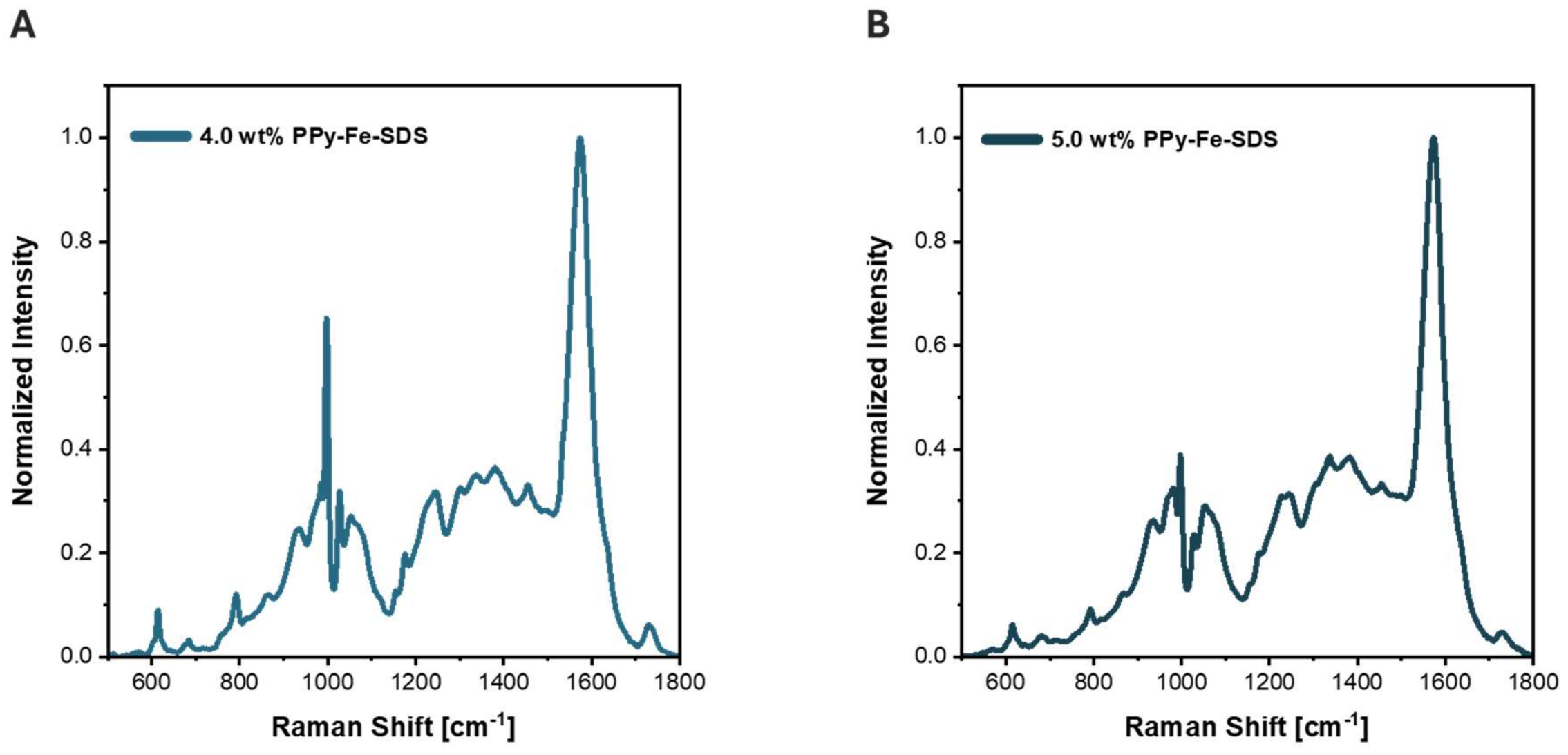

Figure S20: Normalized Raman spectra of single layer printed with inks containing A) 4.0 wt% **PPy-Fe-SDS** and B) 5.0 wt% **PPy-Fe-SDS**.

9. FT-IR Spectroscopic Data of PPy-Fe-SDS Nanocomposites

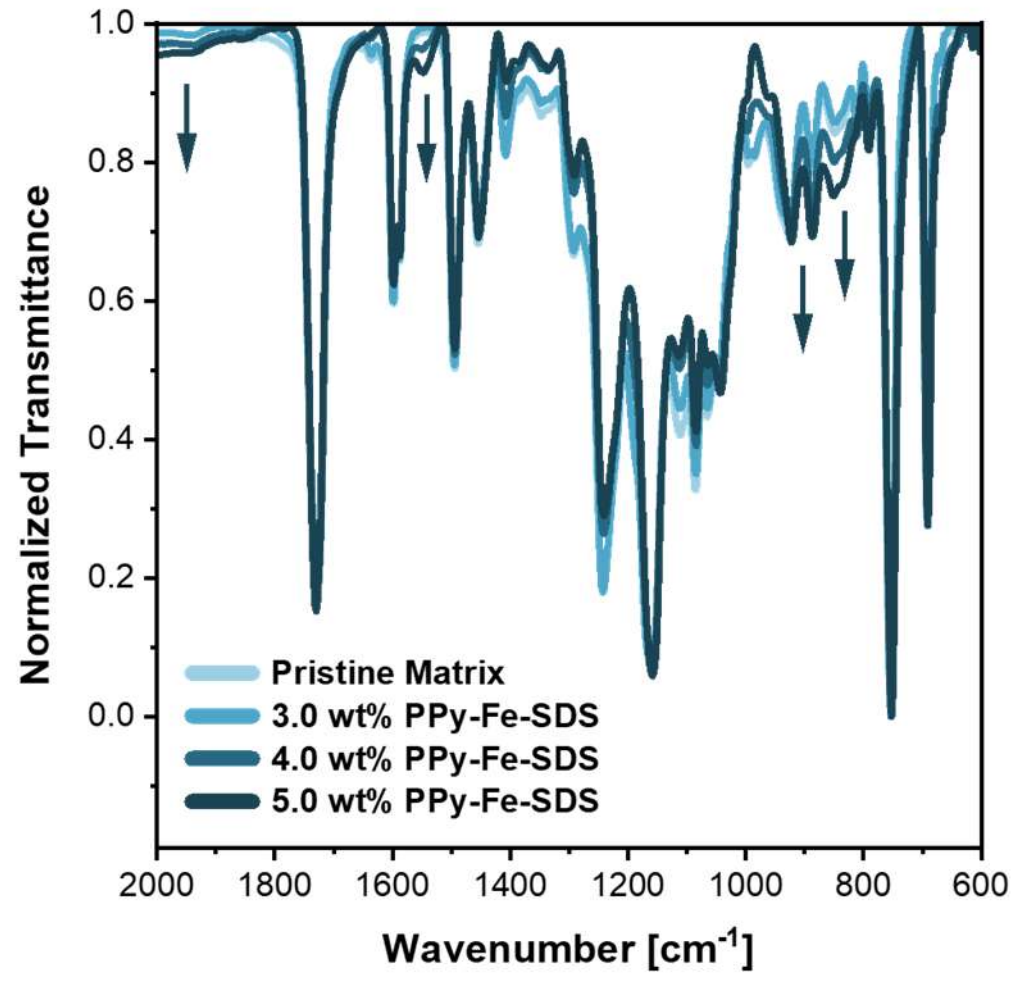

Figure S21: Stacked normalized FT-IR spectra of single layers DLP-printed with 0 – 5.0 wt% of **PPy-Fe-SDS** reveals increased signal intensity for PPy ascribes signals with growing **PPy-Fe-SDS** content.

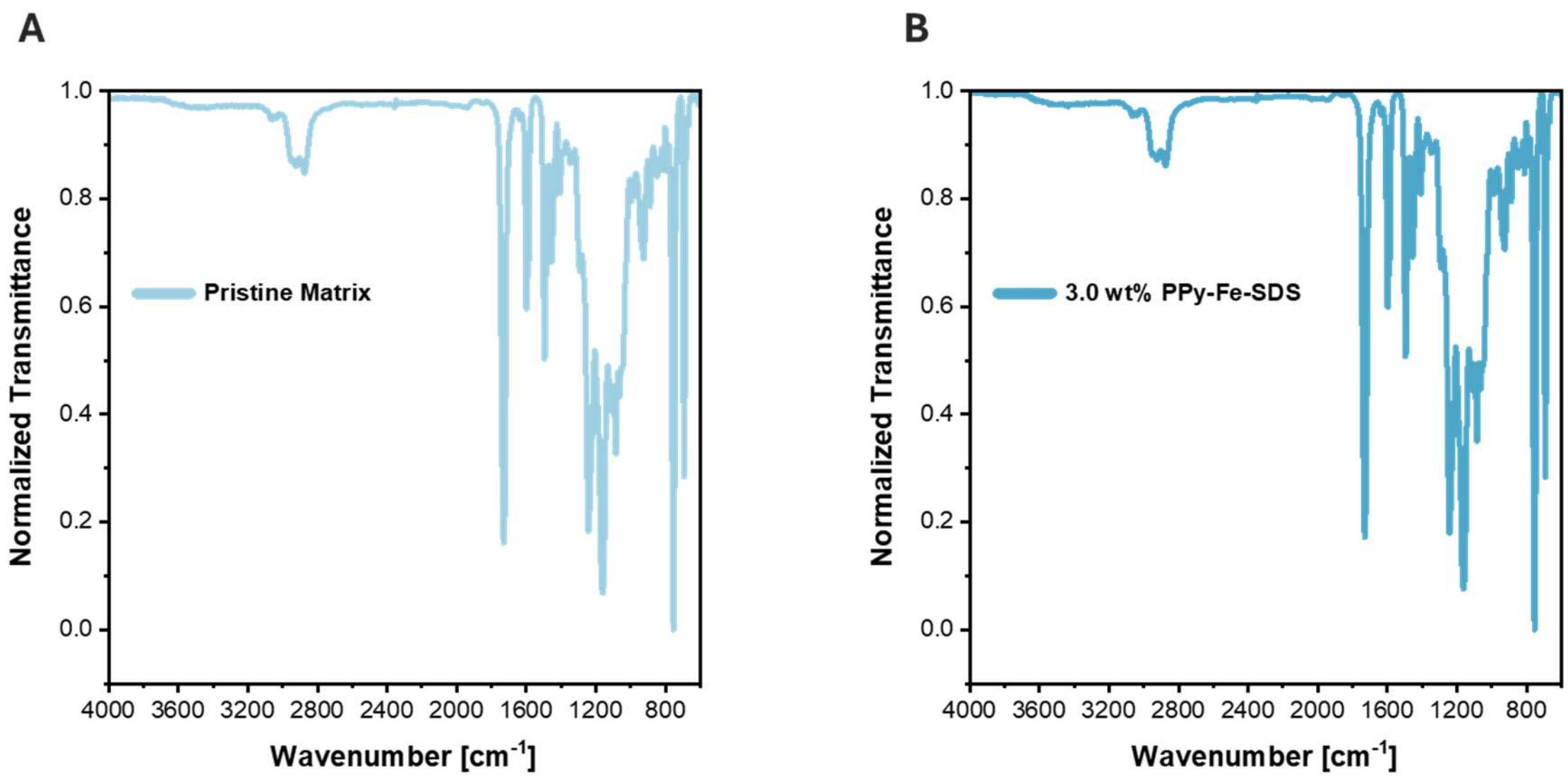

Figure S22: Normalized FT-IR spectra of single layers printed with A) the **pristine matrix** and B) inks containing 3.0 wt% **PPy-Fe-SDS**.

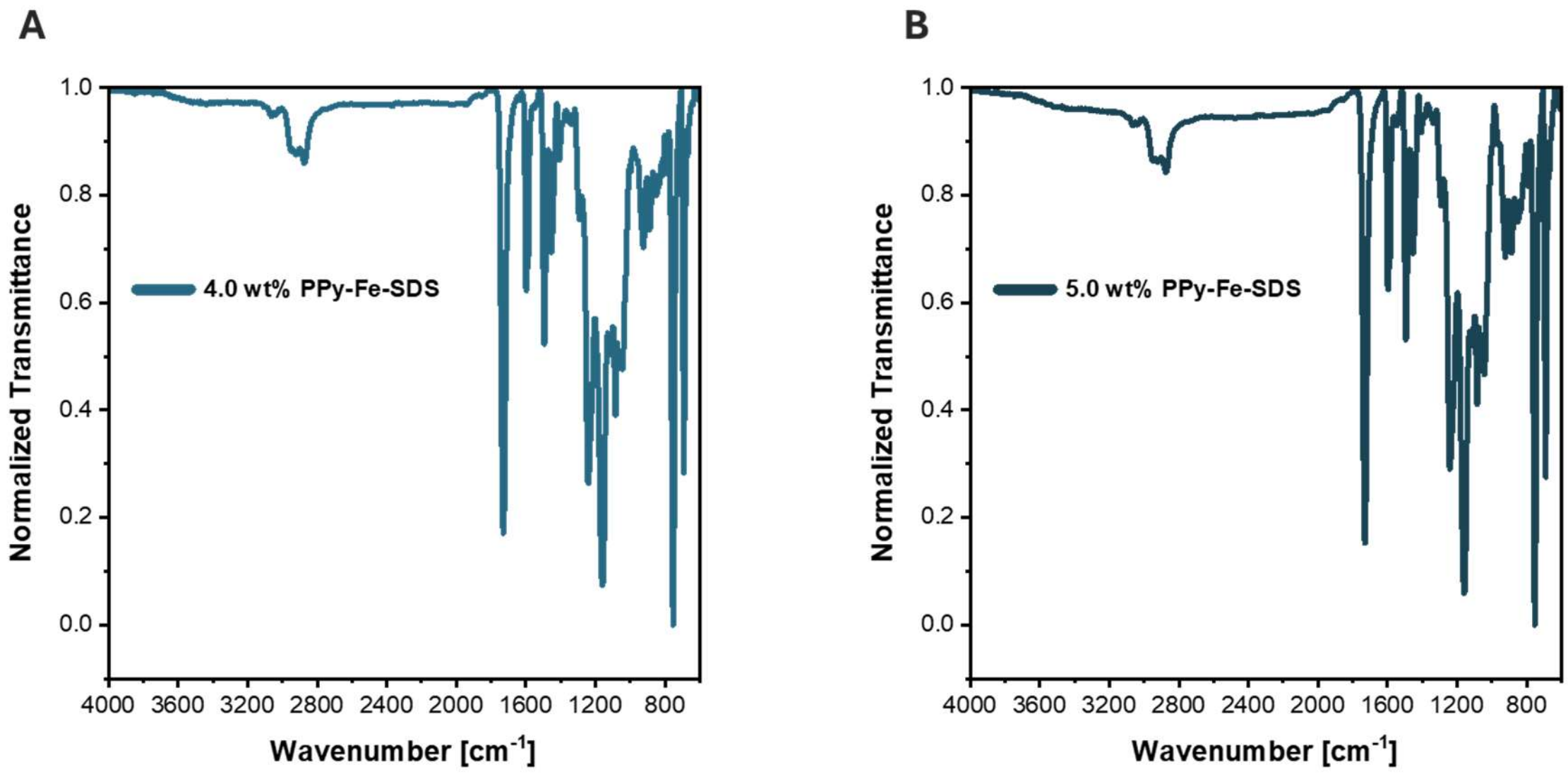

Figure S23: Normalized FT-IR spectra of single layers printed with A) 4.0 wt% of **PPy-Fe-SDS** and B) 5.0 wt% of **PPy-Fe-SDS**.

Double Bond Conversion

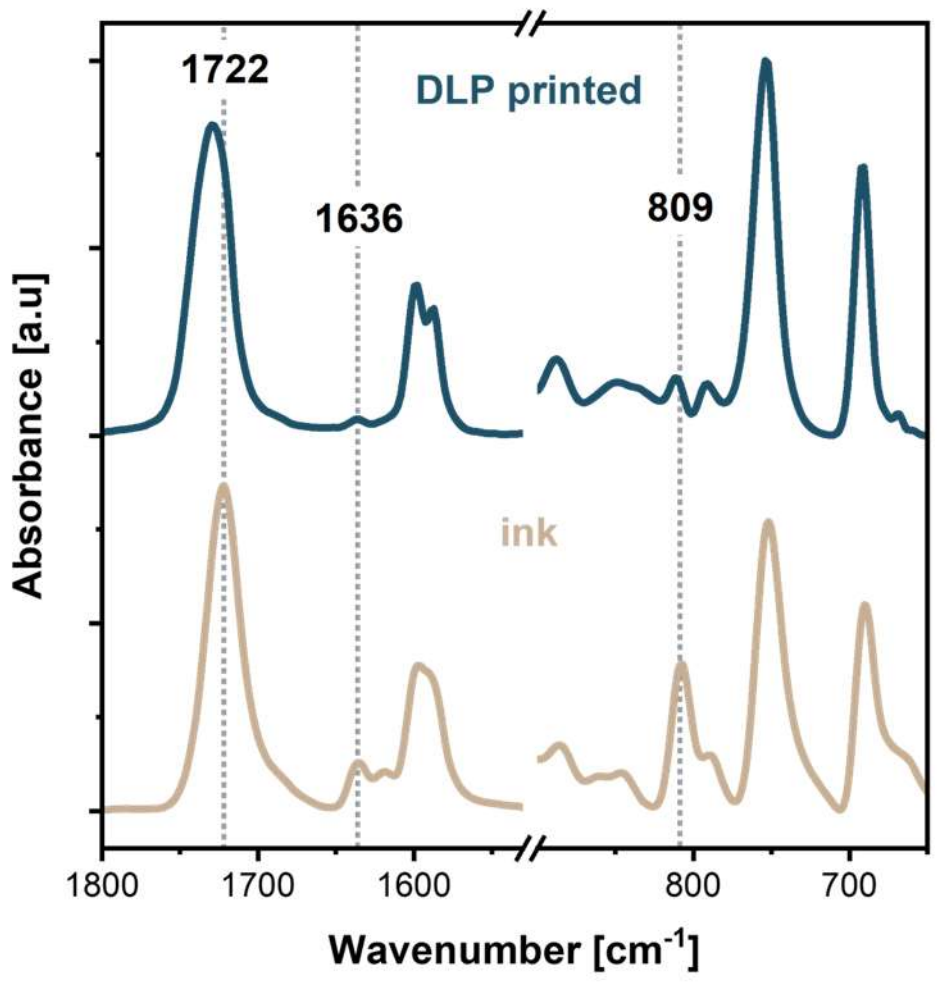

Figure S24: Stacked FT-IR spectra of uncured ink containing 3.0 wt% **PPy-Fe-SDS** and the DLP printed single layer showing decreased C=C stretching (1636 cm^{-1}) and out-of-plane C=C bending (809 cm^{-1}) compared to the C=O stretching (1722 cm^{-1}), (shifted to 1729 cm^{-1} in the printed material).

Table S5: Optimized printing parameters for each ink formulation containing 0 – 5.0 wt% **PPy-Fe-SDS** and corresponding double bond conversion calculated from the comparison of the peak area of the C=O stretching at 1722 cm^{-1} with the C=C stretching at 1636 cm^{-1} and out-of-plane C=C bending at 809 cm^{-1} for uncured ink and DLP printed material, respectively.

Formulation	Exposure Time [s]	Intensity [mW/cm^2]	Double Bond Conversion [%][(a)]
Pristine Matrix	4.9	2.0	88%
3.0 wt% PPy-Fe-SDS	4.0	18	84%
4.0 wt% PPy-Fe-SDS	8.5	18	91%
5.0 wt% PPy-Fe-SDS	30	29	93%

(a) Conversion was averaged using the values obtained for 1636 cm^{-1} and 809 cm^{-1}, respectively.

10. Electrical Conductivities of Nanocomposite Single Layers

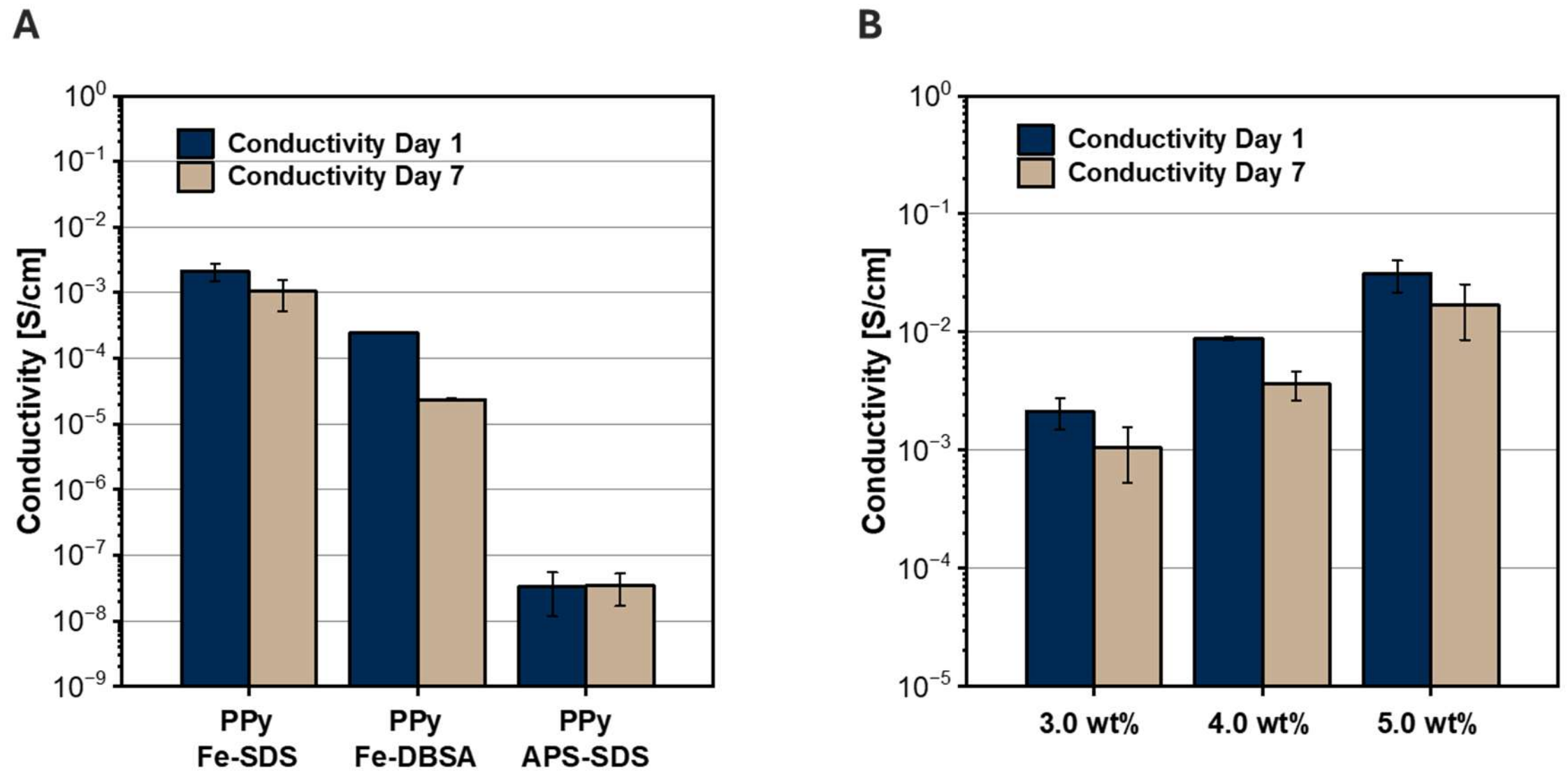

Figure S25: Comparison of electronic conductivity of printed single layers after 1 day and after 7 days using the four-point probe method. All samples were stored under ambient atmosphere after printing. A) Single layers printed with inks containing 3.0 wt% of **PPy-Fe-SDS**, **PPy-Fe-DBSA** and **PPy-APS-SDS**, respectively. B) Single layers printed with inks containing 3.0 – 5.0 wt % **PPy-Fe-SDS**.

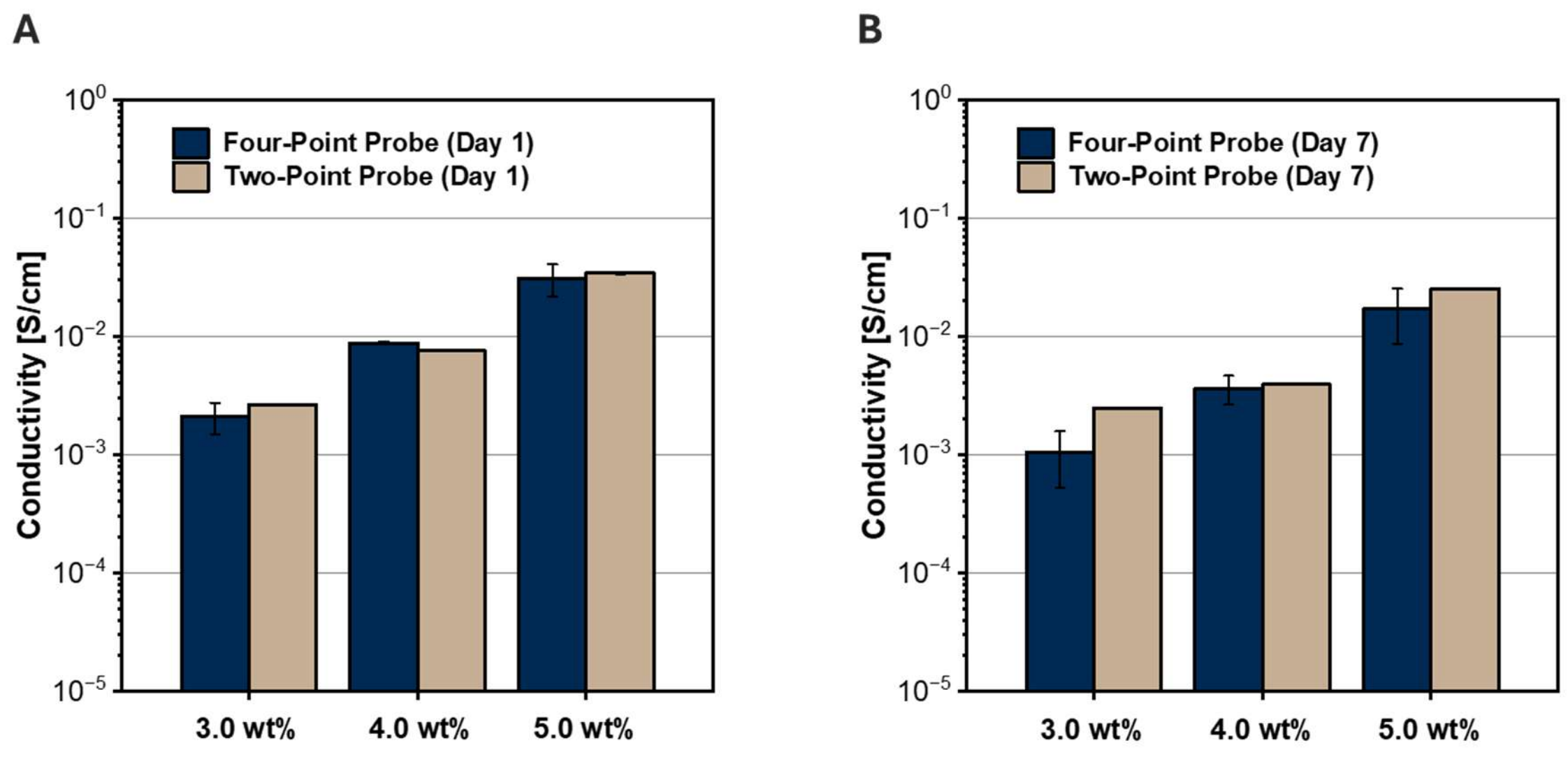

Figure S26: Comparison of electronic conductivity of printed single layers containing 3.0 – 5.0 wt% **PPy-Fe-SDS** comparing the four-point probe method and the two-point probe method. A) Comparison after 1 day. B) Comparison after 7 days.

11. Electrical Impedance Spectroscopy of Nanocomposite Single Layers

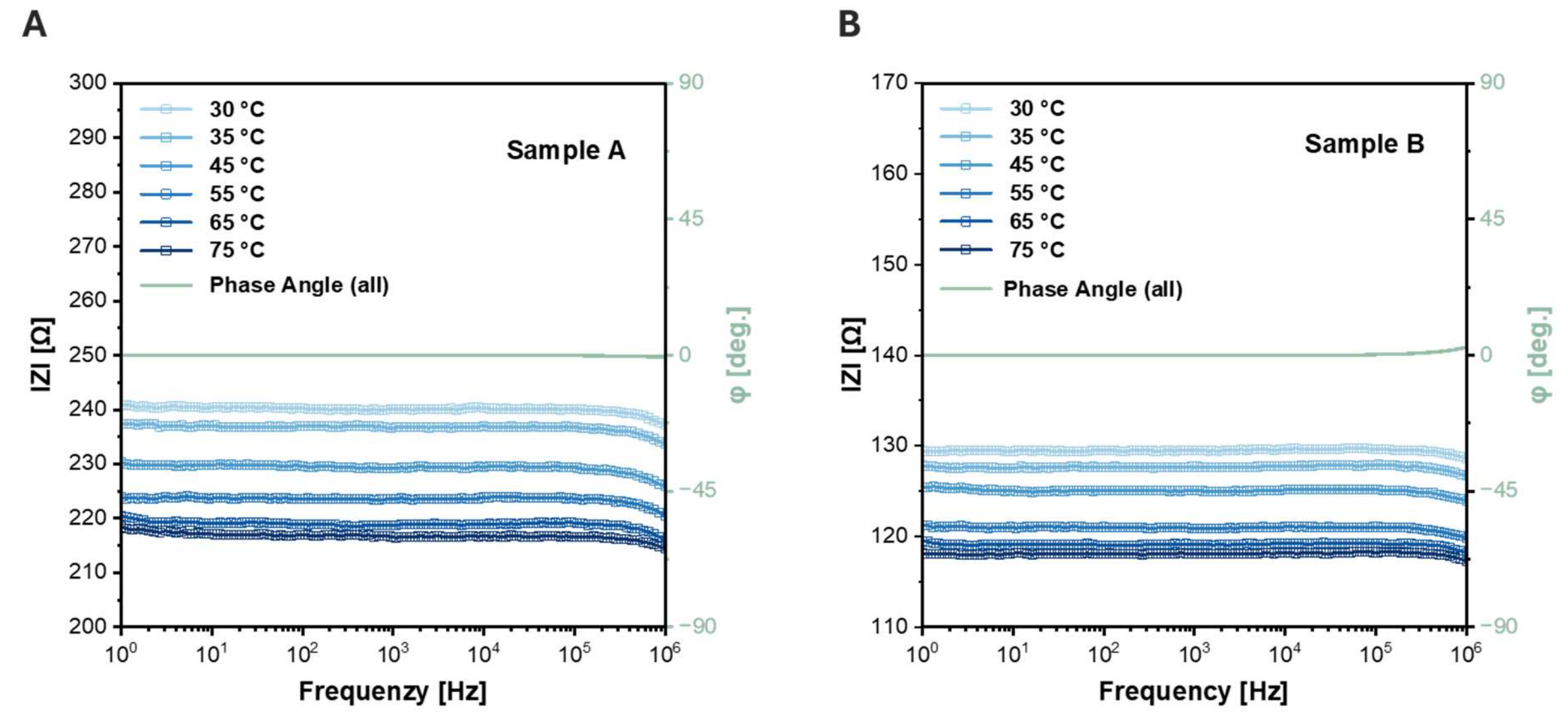

Figure S27: Electrical impedance spectroscopy (EIS) measured on DLP printed single layers containing 3.0 wt% **PPy-Fe-SDS**. Temperature dependent measurements from 30 °C up to 75 °C were conducted after an initial heating to 120 °C for 5 minutes and an additional equilibration time of 5 minutes after temperature change, respectively. (A) **Sample A**. (B) **Sample B**.

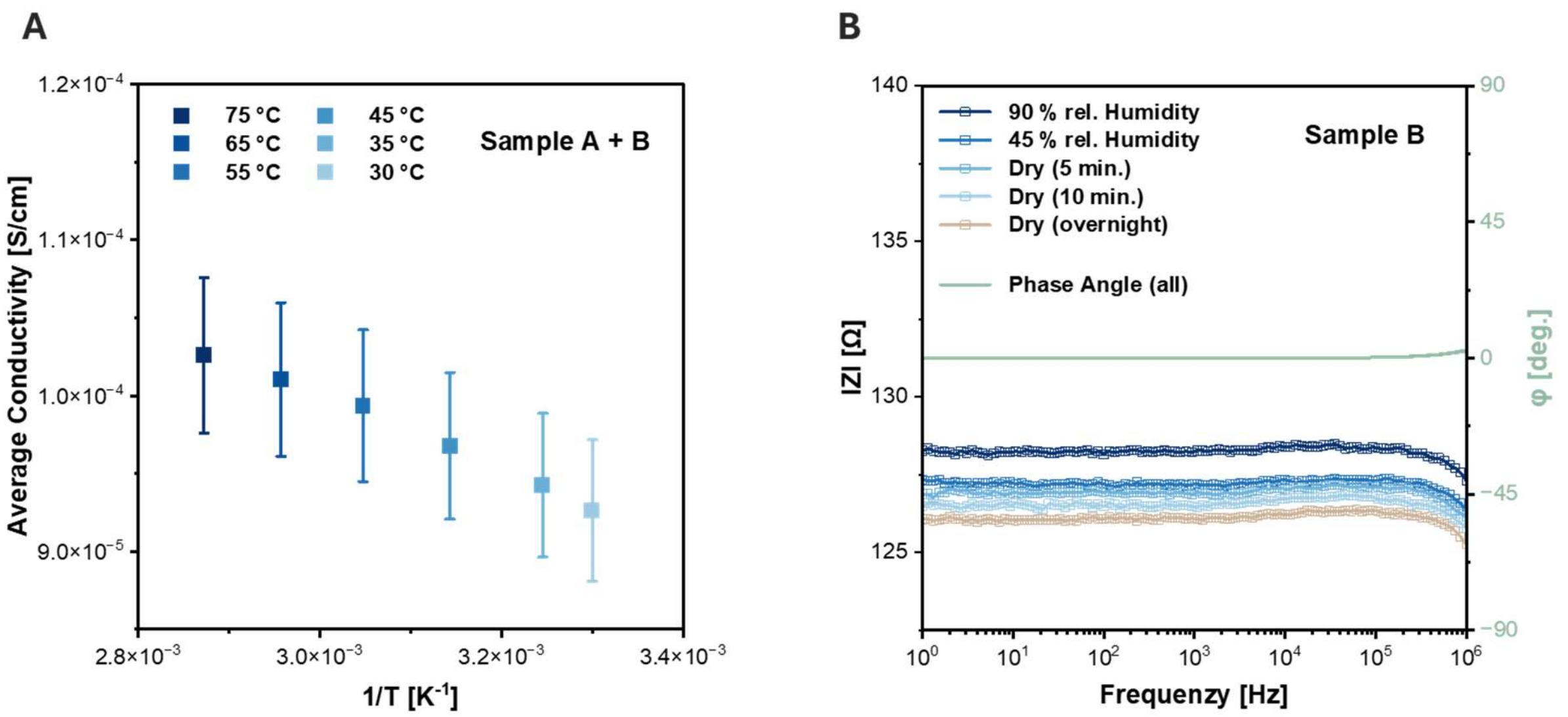

Figure S28: (A) Average electrical conductivity obtained from electrical impedance spectroscopy of **Sample A** and **Sample B** measured at different temperatures. (B) Electrical impedance spectroscopy of **Sample B** at different levels of relative humidity.

12. Comparison with the State of the Art

Table S6: Comparison of own work with state-of-the-art materials containing PPy fabricated by light-based additive manufacturing.

Matrix / Material	PPy Incorporation	Printing Method	Conductivity [S/cm]	Ref.
PNIPAm hydrogel	Post-printing interfacial polymerization of PPy	DLP 3D printing	2.64	49
**Acrylate-based network**	**Pre-synthesised PPy nanoparticles incorporated into the formulation**	**DLP 3D printing**	**3.1×10^{-2}**	**This work**
Metal–organic framework (MOF)	Photo-oxidation of PPy within the MOF	Femtosecond laser direct writing (FsLDW)	2.7×10^{-2}	41
Bisphenol A ethoxylate dimethacrylate (BEMA)	*In situ* photopolymerization of PPy using $AgNO_3$	DLP 3D printing	$< 1.0 \times 10^{-4}$	51
PEGDA hydrogel	Post-printing interfacial polymerization of PPy	DLP 3D printing	7.69×10^{-5}	48
pHEMA-co-MAA NP hydrogel	Pre-synthesised PPy nanoparticles incorporated into the hydrogel	Stereolithography (SLA) printing	5.10×10^{-7}	42
Urethane dimethylacrylate (UDMA)	*In situ* photopolymerization of PPy using $AgNO_3$	DLP 3D printing	$< 1.0 \times 10^{-7}$	50

13. Optical Microscopy Images of PPy-Fe-SDS Nanocomposites

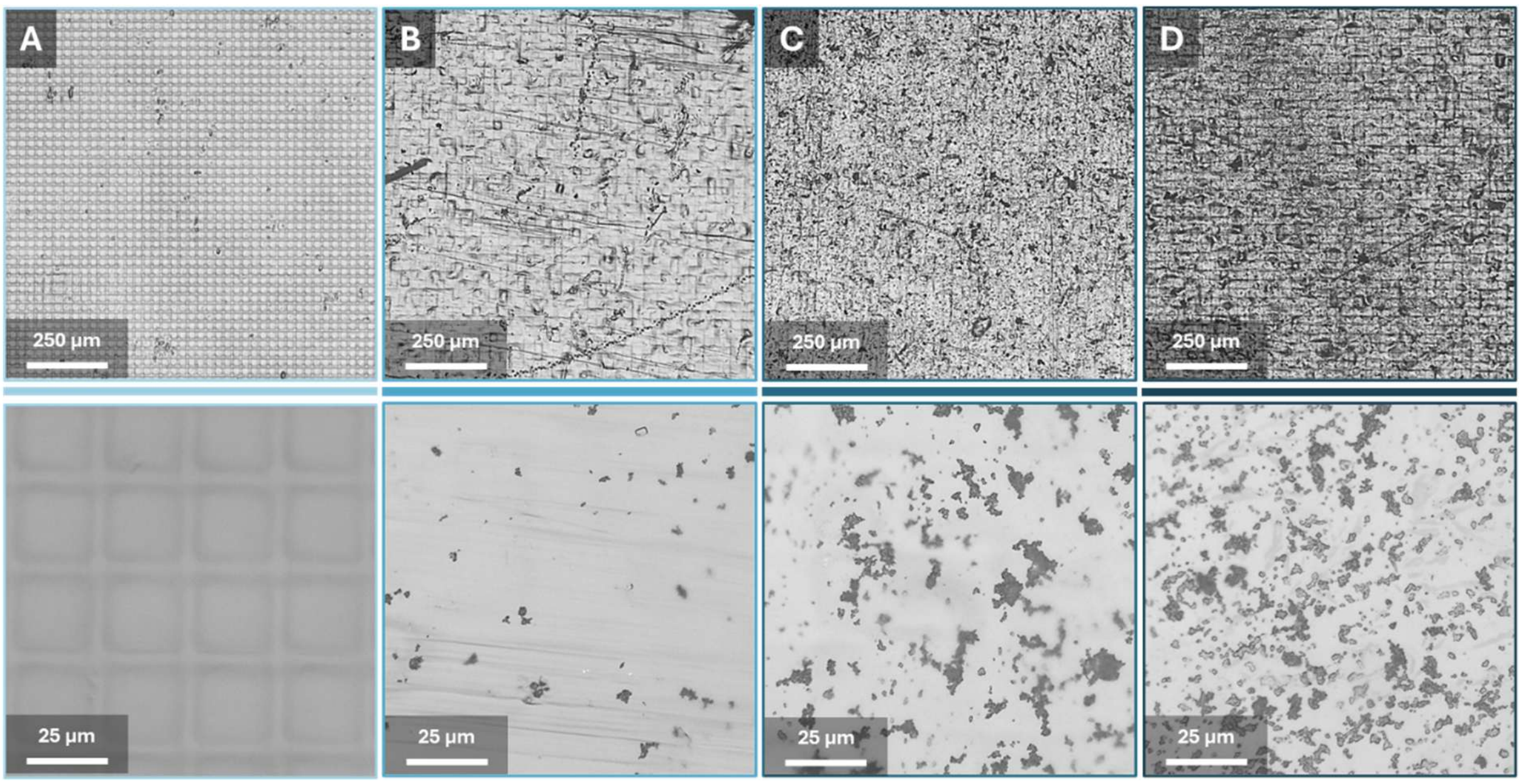

Figure S29: Optical microscopy images of printed single layers using 5× and 50× magnification. The observed grid structure results from the array of single pixels of the DLP printer (25 × 25 µm). With growing content of filler, larger agglomerates are observed. A) **Pristine matrix**. B) 3.0 wt% **PPy-Fe-SDS**. C) 4.0 wt% **PPy-Fe-SDS**. D) 5.0 wt% **PPy-Fe-SDS**.

14. SEM Images of Printed Nanocomposite

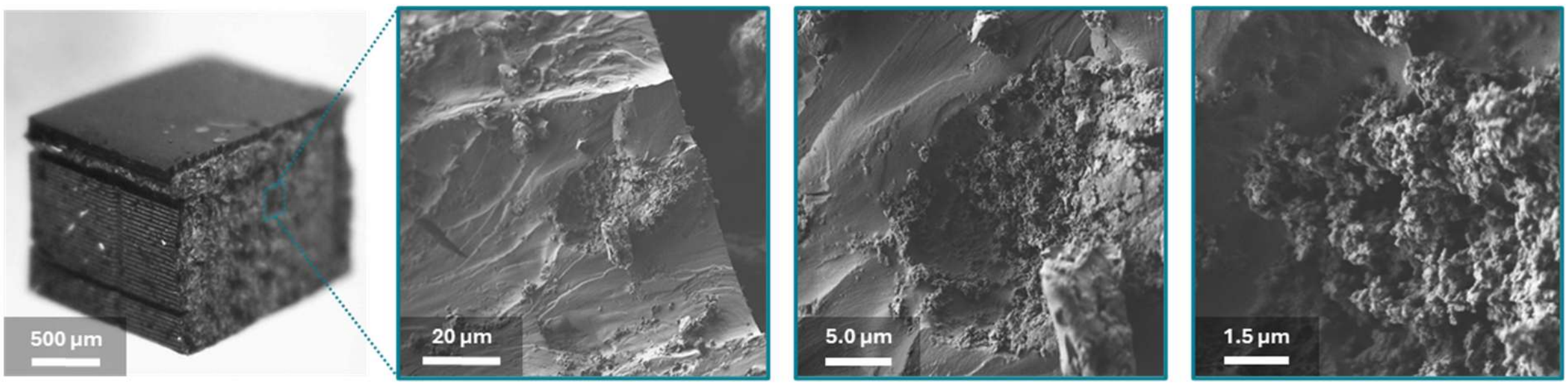

Figure S30: SEM images of the cutting side perpendicular to the layers of a DLP-printed cube using inks containing 3.0 wt% **PPy-Fe-SDS**. Nanoparticles and agglomerates are found and can be identified as **PPy-Fe-SDS** due to the morphology in contrast to the acrylate matrix.

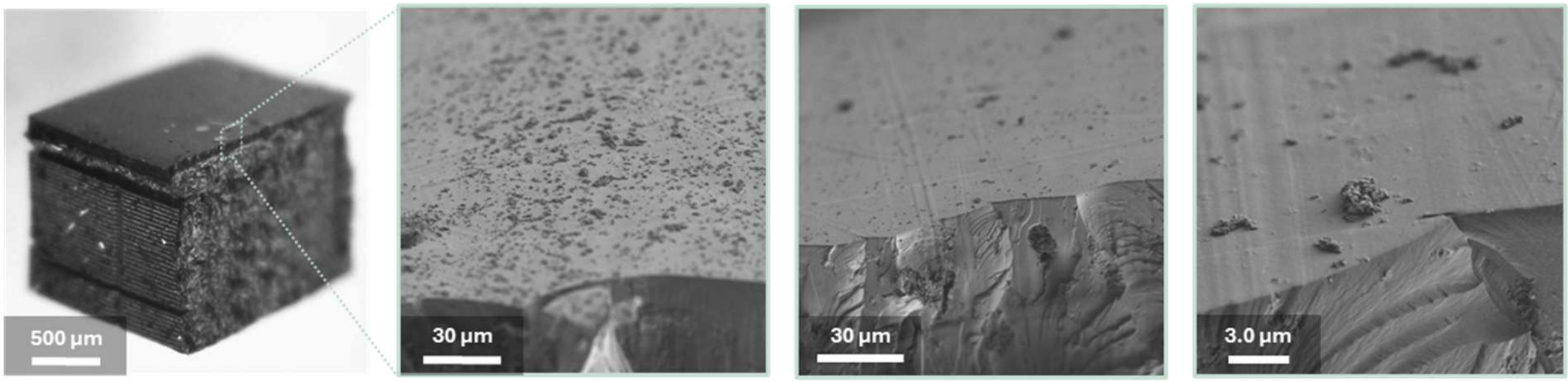

Figure S31: SEM images of the edge between top and cutting side of a DLP-printed cube using inks containing 3.0 wt% **PPy-Fe-SDS**. On the smooth top surface, lower concentration and smaller agglomerates of **PPy-Fe-SDS** are found.

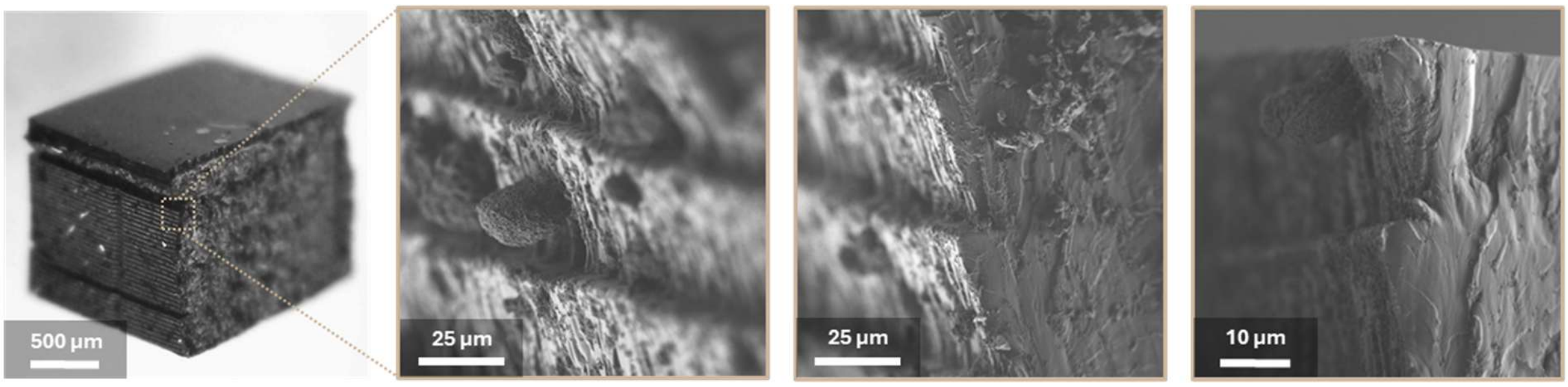

Figure S32: SEM images of the edge between printed layers and the cutting side of a DLP-printed cube using inks containing 3.0 wt% **PPy-Fe-SDS**. Saw-blade profile in the cross-section of the layers is displayed as a result of the light intensity gradient from the illuminated side toward the bottom during curing. The surface is covered in large agglomerates of **PPy-Fe-SDS** decorating the rough surface.

15. Thermogravimetric Analysis Data

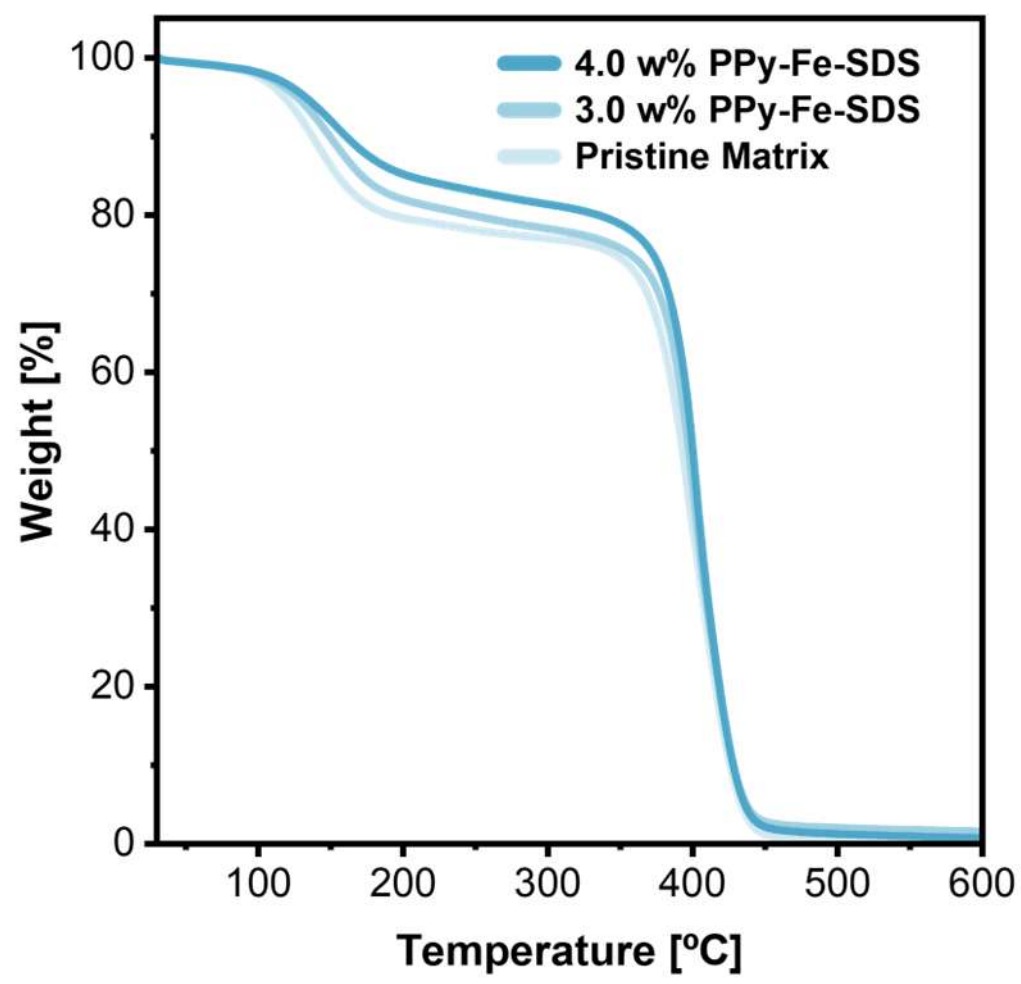

Figure S33: Thermogravimetric analysis of DLP printed cubes (1.0 mm × 1.0 mm × 1.0 mm) comparing materials containing 0 – 4.0 wt% **PPy-Fe-SDS**.

16. Conductivities of Printed Nanocomposite Structures

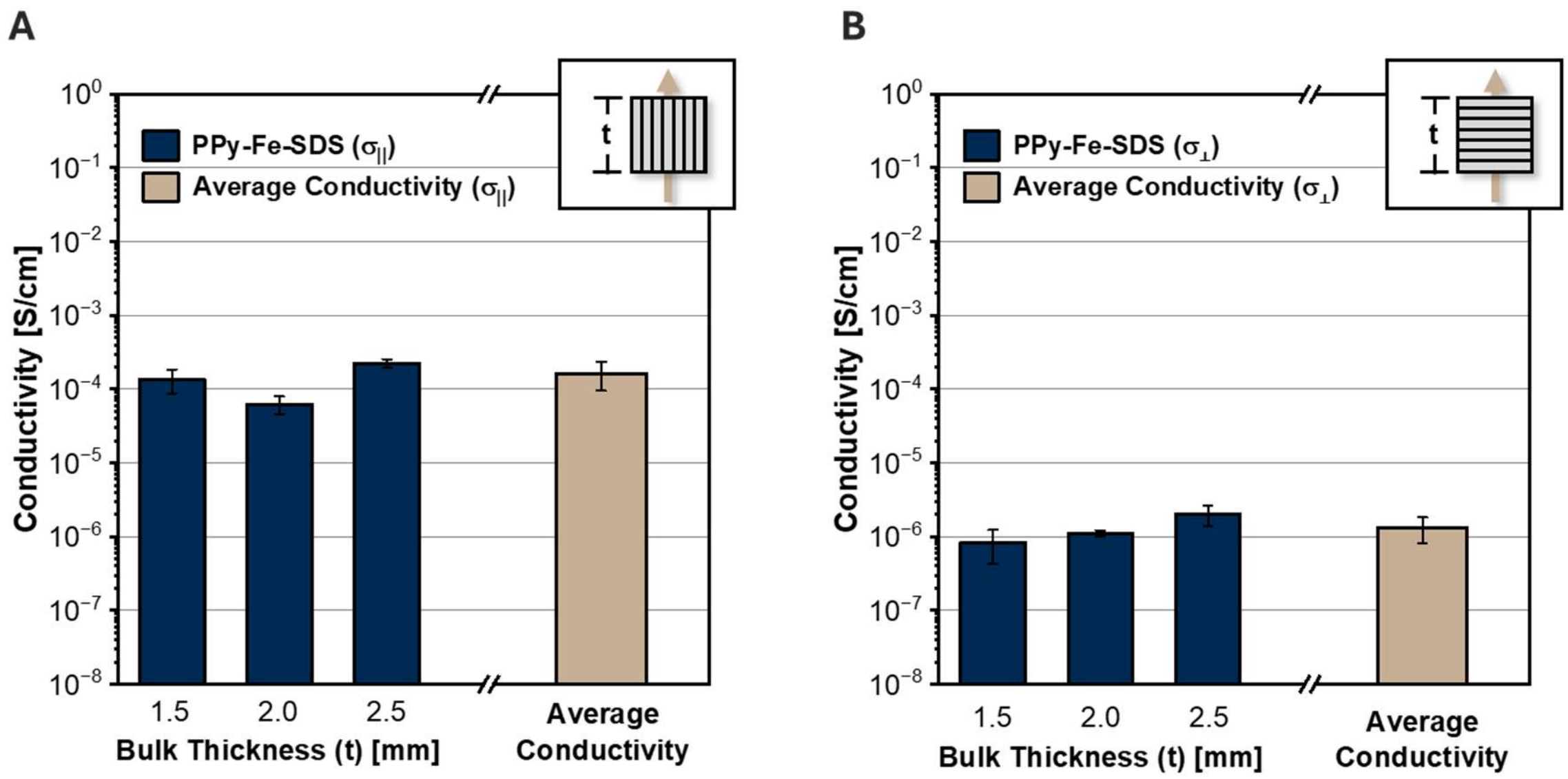

Figure S34: Comparison of conductivity measured parallel and perpendicular to the layers in cubes printed from inks containing 3.0 wt% of **PPy-Fe-SDS**, measured with the two-point probe method. A) Conductivity measurements parallel ($\sigma_{\parallel}$) to the printed layers, comparing three different bulk thicknesses (t) of the printed structure. B) Conductivity measurements perpendicular ($\sigma_{\perp}$) to the printed layers, comparing three different bulk thicknesses (t) of the printed structure.

17. Printability

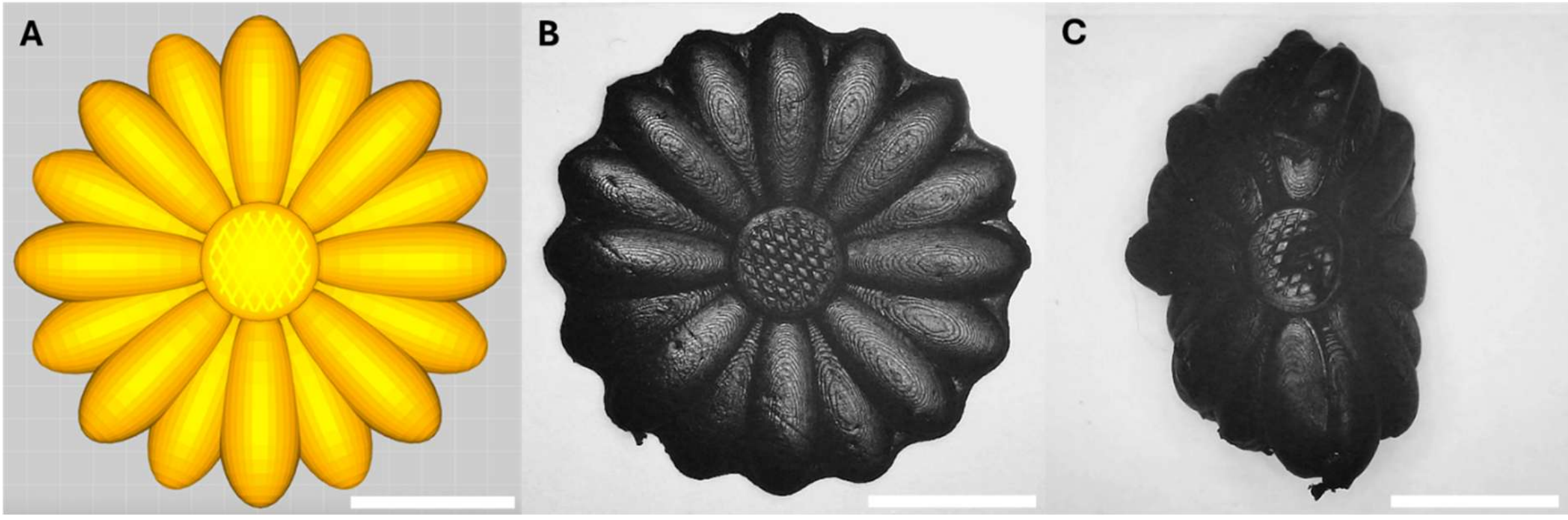

Figure S35: 3D-printed sunflower structure (CAD: 15.0 mm × 15.0 mm × 1.30 mm, measured: 15.2 mm × 15.3 mm × 1.38 mm). Scalebar = 5.0 mm. A) Computational Aided Design (CAD) model. B) DLP-printed structure printed with inks containing 3.0 wt% **PPy-Fe-SDS**. C) Deformed structure printed with inks containing 4.0 wt% **PPy-Fe-SDS**.

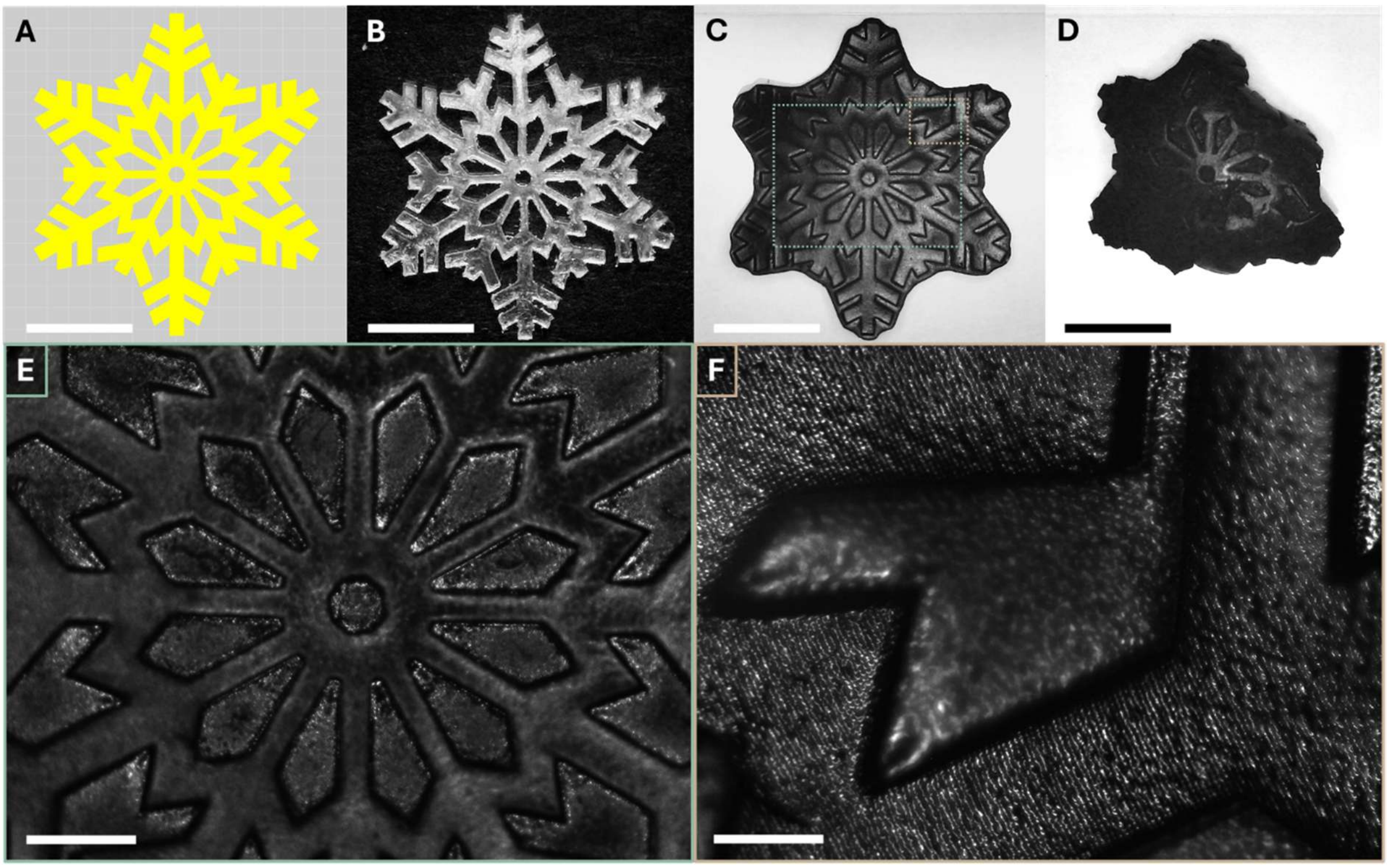

Figure S36: 3D-printed snowflake (CAD: 13.4 mm × 15.0 mm × 0.50 mm, measured: 13.5 mm × 15.3 mm × 0.56 mm) structure. Scalebar: A – D = 5.0 mm, E = 1.5 mm, F = 500 µm. A) CAD model. B) Structure printed with the pristine matrix. C) Same structure printed with inks containing 3.0 wt% **PPy-Fe-SDS**. Due to required burn-in layers, features are covered by overpolymerization. D) Snowflake structure printed with inks containing 4.0 wt% **PPy-Fe-SDS**. E) and F) Magnification of sunflower structure printed with 3.0 wt% **PPy-Fe-SDS**.

18. References

[1] Yussuf A., Al-Saleh, M., Al-Enezi, S., Abraham, G. (2018) Synthesis and Characterization of Conductive Polypyrrole: The Influence of the Oxidants and Monomer on the Electrical, Thermal, and Morphological Properties. *Int. J. Polym. Sci.*, 1–8.

[2] Boukamp, B. A. (1995) A Linear Kronig-Kramers Transform Test for Immittance Data Validation. *J. Electrochem. Soc. 142*, 1885.

[3] Schönleber, M., Klotz, D., Ivers-Tiffée, E. (2014) A Method for Improving the Robustness of linear Kramers-Kronig Validity Tests. *Electrochim. Acta 131*, 20.

[4] Schönleber, M., Ivers-Tiffée, E. (2015) Approximability of impedance spectra by RC elements and implications for impedance analysis. *Electrochem. Commun. 58*, 15.

[5] Schönleber, M., Goyal, R., Ivers-Tiffée, E. *Lin-KK Tool*, KIT.
